# Distributed associations among white matter hyperintensities and structural brain networks with fluid cognition in healthy aging

**DOI:** 10.3758/s13415-024-01219-3

**Published:** 2024-09-20

**Authors:** Marc D. Rudolph, Jessica R. Cohen, David J. Madden

**Affiliations:** 1 Department of Psychology and Neuroscience, University of North Carolina at Chapel Hill, Chapel Hill, NC USA; 2 Biomedical Research Imaging Center, University of North Carolina at Chapel Hill, Chapel Hill, NC USA; 3 Carolina Institute for Developmental Disabilities, University of North Carolina at Chapel Hill, Chapel Hill, NC USA; 4 Brain Imaging and Analysis Center, Duke University Medical Center, Durham, NC USA; 5 Department of Psychiatry and Behavioral Sciences, Duke University Medical Center, Durham, NC USA; 6 Center for Cognitive Neuroscience, Duke University, Durham, NC USA; 7 Present Address: Department of Internal Medicine, Section of Gerontology and Geriatric Medicine, Wake Forest University School of Medicine, Winston-Salem, NC USA; 8 Present Address: Alzheimer’s Disease Research Center, Wake Forest University School of Medicine, Winston-Salem, NC USA

**Keywords:** Cerebrovascular, Subcortical, Structural disconnection, Graph theory

## Abstract

White matter hyperintensities (WMHs) are associated with age-related cognitive impairment and increased risk of Alzheimer’s disease. However, the manner by which WMHs contribute to cognitive impairment is unclear. Using a combination of predictive modeling and network neuroscience, we investigated the relationship between structural white matter connectivity and age, fluid cognition, and WMHs in 68 healthy adults (18–78 years). Consistent with previous work, WMHs were increased in older adults and exhibited a strong negative association with fluid cognition. Extending previous work, using predictive modeling, we demonstrated that age, WMHs, and fluid cognition were jointly associated with widespread alterations in structural connectivity. Subcortical-cortical connections between the thalamus/basal ganglia and frontal and parietal regions of the default mode and frontoparietal networks were most prominent. At the network level, both age and WMHs were negatively associated with network density and communicability, and positively associated with modularity. Spatially, WMHs were most prominent in arterial zones served by the middle cerebral artery and associated lenticulostriate branches that supply subcortical regions. Finally, WMHs overlapped with all major white matter tracts, most prominently in tracts that facilitate subcortical-cortical communication and are implicated in fluid cognition, including the anterior thalamic-radiations and forceps minor. Finally, results of mediation analyses suggest that whole-brain WMH load influences age-related decline in fluid cognition. Thus, across multiple levels of analysis, we showed that WMHs were increased in older adults and associated with altered structural white matter connectivity and network topology involving subcortical-cortical pathways critical for fluid cognition.

**Supplementary information:**

The online version contains supplementary material available at 10.3758/s13415-024-01219-3.

In magnetic resonance imaging (MRI) of the brain, images obtained with a fluid attenuated inversion recovery (FLAIR) sequence often exhibit bright patches. These patches are referred to as hyperintensities and represent fluid-filled pockets of white matter that are the result of cerebrovascular insufficiency (Hachinski et al., 1987; Kertesz et al., 1988; Valdés Hernández et al., 2014; Young et al., 2008). It is thought that these insufficiencies cause restricted blood flow in small vessels (e.g., perforating arteries, arterioles, venules, and capillaries), which contributes to the lesioning of insulated bundles of white matter fibers. This in turn reduces the speed and efficiency of transfer of neural information between brain regions (Li et al., 2018). White matter hyperintensities (WMHs) can be classified into either periventricular (pvWMH) or deep white matter (dWMH) based on their size, continuity, and proximity to the lateral ventricles (Caligiuri et al., 2015; Frey et al., 2019; Kim et al., 2008). Global WMH volume increases with age (Bennett & Madden, 2014; Madden et al., 2017; Raz et al., 2012; Salat, 2011) and has a negative impact on the structural integrity of individual white matter pathways (Bennett & Madden, 2014; Boots et al., 2019; Frey et al., 2019; Quandt et al., 2020; Taylor et al., 2017). In particular, pvWMHs are linked to the degradation of multiple prominent white matter tracts, including the anterior thalamic radiations, forceps minor, and superior longitudinal fasciculi (Acharya et al., 2019; Biesbroek et al., 2013, 2017; Jiang, Paradise et al., 2018; Wen & Sachdev, 2004). That is, while WMHs themselves may be localized, research suggests that WMHs may exert their influence in a distributed fashion and are not constrained by proximity to the site of lesion (DeCarli et al., 1995; Liu et al., 2021; Madden et al., 2017; Wen & Sachdev, 2004).

Age-related changes in brain structure and function, including alterations in brain vasculature, contribute to a decline in speed-dependent (fluid) cognitive abilities involving updating, organizing, and comparing information in working memory (Brugulat-Serrat et al., 2020a, 2020b; Hedden et al., 2016; Jung et al., 2021; Peng et al., 2018; Raz et al., 2012; Shi & Wardlaw, 2016). Fluid cognition is more vulnerable to age-related decline than more knowledge-dependent (crystallized) abilities, such as vocabulary (Craik & Bialystok, 2006; Salthouse, 1996, 2011b). White matter hyperintensities have been linked to poorer cognitive performance on measures of attention (Gunning-Dixon & Raz, 2003; Lockhart et al., 2014, 2015), executive functioning (Birdsill et al., 2014; de Groot et al., 2001; DeCarli et al., 1995; Gunning-Dixon & Raz, 2003; Hoagey et al., 2021), and, to a lesser extent, memory (Acharya et al., 2019; Brugulat-Serrat et al., 2020a, 2020b). One potential mechanism linking WMHs to fluid cognition is their abundance within arterial zones served by the middle cerebral artery and associated lenticulostriate branches that innervate subcortical gray matter regions (Jiang et al., 2018a, 2018b; Wen & Sachdev, 2004). White matter pathways emerging from subcortical gray matter regions, including the basal ganglia and thalamus, have been implicated in fluid cognition (Fama & Sullivan, 2015; Kievit et al., 2016; Ystad et al., 2011) and facilitate both short- and long-range neural communication via cortico-basal-ganglia-thalamocortical systems (Alexander et al., 1986; Pessoa, 2018).

It is increasingly appreciated that the brain’s resilience to pathology lies in the distributed nature of processing, whereby any given pair of regions can be connected by a multitude of pathways (e.g., redundancy). Likewise, cognitive dysfunction is increasingly being characterized in terms of disrupted patterns in both structural (white matter) and functional brain connectivity (Baronchelli et al., 2013; Bressler & Tognoli, 2006; Mill et al., 2017; Reuter-Lorenz & Park, 2014; Sporns, 2014). Recent studies provide evidence that the presence and abundance of WMHs may underlie changes in structural connectivity that contribute to increased disconnection across distributed brain regions in aging (Bennett & Madden, 2014; Langen et al., 2017, 2018; Li et al., 2021; Madden et al., 2017; Thiebaut de Schotten et al., 2020). Specifically, Langen & colleagues (2018) found stronger associations between poorer cognitive functioning and altered structural connectivity based on diffusion-weighted imaging (DWI) between regions whose connecting streamlines passed through voxels classified as WMHs (e.g., the disconnectome) on T2-weighted FLAIR images (Langen et al., 2018). Furthermore, Li & colleagues (2021) used a novel virtual lesioning approach to simulate the impact of pvWMHs, defined by thresholding patterns of hypoperfusion on cerebral blood flow maps obtained via Arterial Spin Labelling MRI, on DWI-based structural connectivity (e.g., the predicted disconnectome; Li et al., 2021). In two separate cohorts (ages 43–56 and 59–88), Li & colleagues showed that the severity of ischemic pvWMHs were associated with reductions in structural connectivity at the group level. Assessing WMH-related disconnection, irrespective of methodological differences, these two novel studies found that subcortical regions were among the most susceptible to the presence of WMHs. Finally, at the network level, WMHs have most commonly been associated with decreased efficiency of information transfer in structural networks based on DWI-based imaging (Boot et al., 2020; Chen et al., 2019; Tuladhar et al., 2016; Yang et al., 2020).

Building on this foundational work, the present study sought to further assess the extent to which the location of WMH deposition relates to the age-related disruption of short- and long-range communication patterns in a sample of adults ages 18 − 78. Our primary goal was to characterize associations between chronological age, WMHs, cognition, and DWI-based structural connectivity without imposing external constraints on connectome construction, thus leveraging our imaging data to the fullest extent possible. The present study combined predictive modeling and network-based analyses to assess brain-wide associations between age, fluid cognition, WMHs, and structural connectivity derived by using DWI. We tested the hypothesis that structural connectivity decreased with age, particularly due to disruption of subcortical-cortical pathways, and that this was associated with the presence of WMHs. Furthermore, we hypothesized that these age-related differences in structural connectivity were associated with reduced fluid cognition.

## Materials and methods

### Participants

This research was conducted in accordance with the Code of Ethics of the World Medical Association (Declaration of Helsinki) for experiments involving humans. The protocol was approved by the Duke University Institutional Review Board, and participants gave written informed consent at the start of the study. Behavioral and neuroimaging data were collected from 68 cognitively normal community-dwelling adults between 18 and 78 years of age. All participants reported that they possessed at least 12 years of education and completed a health questionnaire to assess significant health problems (Christensen et al., 1992). All participants reported being free from significant health problems (including atherosclerosis, neurological, and psychiatric disorders). Individuals were excluded if they reported a significant history of stroke or cardiovascular disease, other than treated hypertension, or current medication for cardiovascular disease. Merenstein et al. (2023) report additional details regarding this sample and exclusion criteria. That manuscript reported fMRI activation data and did not report any of the WMH data, which are the focus of this report. Ages for the sample of 68 participants were distributed across younger (18–39 years; *n* = 28; *M* = 28.04; *SD* = 5.27), middle-aged (40–59 years; *n* = 20; *M* = 48.75; *SD* = 5.80), and older adult (60–78 years; *n* = 20; *M* = 69.15; *SD* = 4.61) cohorts. Age was treated as a continuous measure in all analyses. Characteristics of the final sample are presented in Table 1.

**Table 1:** Participant characteristics

	*M*	*SD*	*r* with age
Education (years)	17.412	2.294	0.006
MMSE	29.588	0.604	0.004
Vocabulary	58.529	4.530	− 0.018
BDI	2.647	3.203	0.077
Color vision	13.875	0.476	− 0.252*
Visual acuity (log MAR)	− 0.009	0.085	0.355**
General fluid cognition^†^	0.0	0.969	− 0.859***
Perceptual speed^†^	0.0	0.947	− 0.808***
Executive function^†^	0.0	0.795	− 0.784***
Memory^†^	0.0	0.810	− 0.679***

Age was treated as a continuous measure in all analyses. Characteristics of included participants (*n* = 68). MMSE = raw score on the Mini-Mental State Exam (Folstein et al., 1975); Vocabulary = raw score on the vocabulary subtest of the Wechsler Adult Intelligence Scale III (Wechsler, 1997); BDI = total score on the Beck Depression Inventory (Beck et al., 1961); Color Vision = score on Dvorine color plates (Dvorine, 1963); Visual Acuity = logarithm of the minimum angle of resolution (MAR) for the Freiburg Visual Acuity Test (Bach, 1996); Log MAR of 0 corresponds to Snellen 20/20, with negative values corresponding to better resolution. Thus, the positive correlation for acuity represents age-related decline in this measure. General Fluid Cognition = factor score for 12 reaction time and psychometric tests sampling the three cognitive domains of perceptual speed, executive function, and memory, with four tests per domain (see *Cognitive Measures*). Perceptual Speed = factor score for reaction time and psychometric tests of perceptual speed. Executive Function = factor score for reaction time and psychometric tests of executive function. Memory = factor score for reaction time and psychometric tests of memory. Factor-score correlations with age are covaried for sex and WAIS vocabulary. *p < 0.05 (uncorrected), **p < 0.01 (uncorrected), ***p < 0.001 (uncorrected)

### Cognitive measures

Participants completed a cognitive battery of 12 neuropsychological tests covering three domains: perceptual speed, executive function, and memory. Each domain included three computer-based tests of reaction time (RT), as described by Merenstein et al. (2023), and one test from the cognition section of the NIH Toolbox (Gershon et al., 2013). Tests for perceptual speed included (1) simple RT, pressing the space bar at the onset of a square; (2) choice RT for a left- or right-facing arrow; (3) choice RT for the displayed color of a non-color word; and (4) number correct in 85 s from the NIH Toolbox Pattern Comparison Test. Executive function tasks included (1) a digit-symbol coding task (Salthouse, 1992); (2) flanker task interference (incompatible RT divided by compatible RT); (3) Trails B minus Trails A (Reitan, 1992); and (4) the computed score on the NIH Toolbox Dimensional Change Card Sort Test. Memory tasks included (1) the Wechsler Adult Intelligence Scale III (WAIS-III) logical memory subtest (Wechsler, 1997); (2) the California Verbal Learning Test delayed memory subtest (Delis et al., 2004); (3) a visual working memory task (Saults & Cowan, 2007); and (4) the computed score for the NIH Toolbox Picture Sequence Memory Test.

A factor-analytic approach was used to estimate general domain-specific measures of perceptual speed, executive function, and memory (Hedden et al., 2012, 2016; Madden et al., 2017, 2020). From these estimates, a general summary measure of fluid cognition was obtained by taking the first unrotated factor from the principal axis factor analysis, controlling for WAIS-III vocabulary and sex. Statistical analyses were conducted by using SAS 9.4 (SAS Institute, Inc., Cary, NC) within the general linear model. Cognitive testing procedures and details regarding the factor analysis are provided in greater detail elsewhere (Madden et al., 2017, 2020). Secondary analyses indicated that, for these data, the individual cognitive domains of perceptual speed, executive function, and memory did not account for a significant portion of age-related variance, independently of their shared variance across the 12 tests. We therefore used an overall measure of general fluid cognition (first factor of the 12 tests) as the primary cognitive variable in all primary analyses. In supplementary analyses, we explored the relationship between age and WMH load with three individual measures selected from the NIH toolbox indexing the cognitive domains of executive function, memory, and perceptual speed as described above.

### MRI data acquisition

Imaging data were collected on a 3T GE MR750 whole-body 60 cm bore MRI scanner (GE Healthcare, Waukesha, WI) equipped with 50 mT/m gradients and a 200 T/m/s slew rate. The scanner possessed a 48-channel head coil that was used for radio frequency (RF) reception. Participants wore earplugs to reduce scanner noise, and foam pads were used to minimize head motion. Three-plane (straight axial/coronal/sagittal) localizer fast spin-echo images were acquired at the start of the scan to define the volume for data collection. Global field homogeneity was ensured using a semiautomated high-order shimming program. Anatomical T1-weighted images included 292 straight axial slices attained using a 3D fast inverse-recovery-prepared spoiled gradient recalled (SPGR) sequence with repetition time (TR) = 2203.5 ms, echo time (TE) = 3.076 ms, inversion recovery time (TI) = 900 ms, field of view (FOV) = 240 mm × 240 mm, flip angle = 8°, voxel size = 0.47 × 0.47 × 0.5 mm, 512- × 512-mm acquisition matrix, and a sensitivity encoding (SENSE) factor of 2, using the array spatial sensitivity encoding technique and extended dynamic range. T2-weighted DWI images were acquired with 232 contiguous slices with TR = 6300 ms, TE = 105 ms, FOV = 256 mm^2^, flip angle = 90, voxel size = 1 × 1 × 1.4 mm, 256 mm^2^ acquisition matrix, and a SENSE factor of 1. Slice orientation was axial oblique, parallel to the AC-PC plane. The DWI data consisted of 92 contiguous slices acquired along the AC-PC plane using dual-echo, spin-echo parallel EPI pulse sequence, with TR = 4894 ms, TE = 64.7 ms, FOV = 220 mm^2^, flip angle = 90°, voxel size = 1.5 mm^3^, acquisition matrix size = 144 mm^2^, and SENSE acceleration factor of 1. The multishell DWI acquisition protocol collected 90 diffusion-encoding directions, two b values (b-value 1 = 1500 s/mm^2^; b-value 2 = 3000 s/mm^2^), and two high-resolution nondiffusion-weighted b0 (0 s/mm^2^) shells. The scanning session also included one run of resting-state functional MRI, four runs of task-related functional MRI, and one run of susceptibility-weighted structural imaging that were not used in the current analyses and are thus not discussed here.

### White matter hyperintensity classification and subcortical volume estimation

Global WMH volumes (i.e., including both pvWMH and dWMH) were calculated with the unidentified bright object (UBO) detector (Jiang et al., 2018a, 2018b), a fully automated machine-learning toolbox. High-resolution T1-weighted images were used to facilitate detection of WMHs. T1-weighted images were segmented into gray matter, white matter, and CSF tissue-types and registered to one of three age-specific templates: < 55 (used for participants 18–55), 56–75 (used for participants 60–75), and 70–80 years of age (used for participants aged ≥ 75 years) (Jiang et al., 2018a, 2018b) using diffeomorphic anatomical registration through exponentiated lie (DARTEL) space (Ashburner, 2007). In DARTEL space, nonbrain tissue was removed from the T1-weighted and FLAIR images before construction of gray matter, white matter, and CSF tissue probability maps using FMRIB’s automated segmentation tool (FAST) (Zhang et al., 2001). T2-weighted FLAIR images were coregistered to T1-weighted images. K-nearest neighbors (k-NN) clustering, a supervised machine-learning algorithm, was then used to classify white matter voxels that appear hyperintense on T2-weighted FLAIR images and hypointense on T1-weighted images (for a stepwise description of all pre- and post-processing steps; see Jiang et. al., 2018). A default setting of 12 mm from the lateral ventricles was used to further delineate periventricular from deep WMHs (Jiang et al., 2018a, 2018b). The probability, *k,* of classifying voxels as WMH in reference to the provided training dataset was set at the recommended default *k* = 0.7 and *k* = 0.5. Multiple thresholds were assessed to ensure effects were not driven by the selected threshold. To assess detection of sparse punctate lesions in our normative sample, we also calculated WMHs at *k* = 0.5. To capture the total effects of WMH load on structural brain network topology and communication capacity, we focused on global WMH for all primary analyses. However, we also estimated and summarized pvWMH and dWMH separately (see *Statistical Analyses*). WMH load was further delineated by arterial and tract-specific derivations to test our hypothesis that the relationship between increased WMHs and decreased connectivity of structural brain networks involves the disruption of subcortical-cortical pathways (see Supplementary Material and Fig. S1). As described by Jiang et al., (2018a, 2018b), the UBO toolbox applies both arterial and tract-specific masks to every individual’s whole-brain WMH map in DARTEL space. Arterial territories were manually delineated on a single human brain imaged with computed tomography and MRI as described by Wen and Sachdev (2004; see Supplementary Methods for additional details).

### DWI processing

We processed the DWI data with MRtrix3 (Tournier et al., 2019), first resampling to an isotropic voxel size of 1 mm^3^, using the MRtrix3 *mrgrid* function. We then de-noised with MRtrix3 by using the function *dwidenoise* and preprocessed using the MRtrix3 *dwifslpreproc* function. Head motion, eddy currents, and susceptibility-induced distortions were corrected with FSL’s EDDY tool. Outlier estimation and replacement were specified via the -repol option, and off-resonance field distortions were corrected by applying FSL TOPUP (Andersson et al., 2003). Quality control reports were generated for all participants by specifying the FSL *eddy_quad* option (Bastiani et al., 2019). Total head motion estimates represent the average voxelwise displacement (mm) across volumes and are a summary of rotational and translational movement around the x, y, and z axes. Absolute motion estimates (*M* = 0.475 mm; *SD* = 0.099) were calculated with respect to a reference frame (average b0 image). Relative, or framewise motion estimates (*M* = 0.147; *SD* = 0.048), were calculated based on volume-to-volume movement. All motion estimates were well below those reported by Bastiani & colleagues (2019). The DWI bias-field correction and intensity normalization were completed with the MRtrix3 functions *dwibiascorrect* and *mtnormalise* respectively. Next, tissue-specific response functions for white matter, gray matter, and cerebrospinal fluid (CSF) were estimated via the *dwi2response* command and were used to generate fiber orientation distribution (FOD) maps. The FOD maps were constructed via the *dwi2fod* command by using multishell multitissue constrained spherical deconvolution using the *mmst_csd* algorithm (Tournier et al., 2012). Anatomically constrained tractography (ACT) was adopted to define the interface between white matter and gray matter. To generate the gray matter/white matter boundary used for seeding of streamlines, we registered the high-resolution anatomical T1 image to diffusion space for each individual and segmented the image into five tissue types: cortical gray matter, subcortical gray matter, white matter, CSF, and pathological tissue.

#### **Structural white matter connectivity**

Following segmentation, we used probabilistic tractography to generate streamlines via the *tckgen* command. The -act and -backtrack options were specified to anatomically constrain the extent of streamline propagation and ensure proper termination of streamlines in the gray matter/white matter interface. This ensures the biological plausibility of generated streamlines across individuals. For each participant, 10 million streamlines were generated and iteratively filtered by a factor of 10 (Tournier et al., 2019) to 1 million streamlines via spherical-deconvolution informed filtering of tractograms (SIFT) in MRtrix3 (Smith et al., 2013), via the *tcksift* command. SIFT identifies false-positive streamlines that do not match the underlying white-matter anatomy via minimization of a cost function based on the FOD (Yeh et al., 2016). Structural connectomes were generated from the filtered streamlines via the *tck2connectome* command. Specifically, we defined 274 regions of interest (ROIs) by using the multimodal human Brainnetome atlas comprising 210 cortical, 36 subcortical, and 28 cerebellar regions (https://atlas.brainnetome.org/bnatlas.html) (Fan et al., 2016; Sang et al., 2012). Regions were registered to a standard template in MNI space via FSL’s FLIRT (FMRIB's linear image registration tool) (Smith et al., 2001) and then mapped to an individual participant's native space. Because of incomplete coverage, cerebellar regions were excluded, leaving a total of 246 cortical and subcortical ROIs. Symmetrical 246 × 246 structural connectivity matrices (weighted; undirected) were constructed whereby each cell of the matrix represented the total number of streamlines propagating through a pair of ROIs. Only cells containing three or more streamlines were retained for analyses, and cells with fewer than three streamlines were set to zero. Finally, structural connectivity matrices were corrected to account for variation in ROI volumes by specifying the *-scale_invnodevol* option for the MRtrix3 *tck2connectome* command (Hagmann et al., 2008).

In addition to the subcortex and cerebellum, regions of the Brainnetome atlas are labeled by anatomical location and grouped into frontal, temporal, parietal, insular, limbic, and occipital lobes. The limbic lobe, as defined by the Brainnetome atlas, consists solely of regions within the cingulate gyrus. In addition to anatomical location, we assigned each of the cortical regions in the Brainnetome atlas to one of seven functionally defined intrinsic networks (Yeo et al., 2011): the Default Mode (DMN), Dorsal Attention (DAN), Frontoparietal (FPN), Limbic (LN), Somatomotor (SMN), Ventral Attention (VAN), and Visual (VIS) networks. Using this parcellation, we also assigned subcortical nodes to an eighth (SUB) network.

### Structural network topology and graph theoretical metrics

Graph theoretical metrics that summarize structural brain network organization were computed from each individual’s weighted structural connectivity matrix. Specifically, we computed density, clustering coefficient, modularity, and communicability. Density quantifies the degree of connectedness for a given graph and is quantified by relating the number of edges to the total possible edges. Clustering coefficient measures the degree of connectedness between neighboring nodes by calculating the probability that two neighbors of a node are themselves connected. Modularity represents the degree to which a system or network can be partitioned into distinct modules, or networks, with high interconnectedness within each module. Finally, communicability quantifies the total number of paths, direct or indirect, that can be traversed by a random walker between any pair of regions. Together, these metrics assess aspects of general network structure and information transfer. Each metric is formally defined, along with their corresponding equations, in the *Supplementary Methods*.

## Statistical analyses

### Linear relationships between age, fluid cognition, and WMHs

Pearson’s correlations were conducted between age, fluid cognition, and WMHs (global WMH, pvWMH, and dWMH) using the *corr* function in *R* (R Core Team, 2019). Power analyses (Faul et al., 2007) indicated that with 68 participants, a Pearson correlation r value of 0.35 (a medium effect-size) would be detected as significant at alpha = 0.05 (two-tailed), with a power of 0.84. Correlations between age, fluid cognition, and WMHs on arterial and tract-based levels are described in *Supplementary Results*. Additionally, a partial correlation between global WMH and fluid cognition was conducted controlling for age. Because the age-related increase in WMH volume is nonlinear, typically increasing more at later ages, WMH estimates were log-transformed before all analyses, consistent with prior work (Madden et al., 2017; Raz et al., 2005, 2012). Correlations were corrected for multiple comparisons using the false discovery rate (FDR) (Benjamini & Hochberg, 1995).

To ensure that findings could not be attributed to motion or data quality, in supplementary analyses, we assessed relationships between age, fluid cognition, and global WMHs with quality control metrics estimated during DWI preprocessing, including (1) average absolute motion; (2) average relative motion; (3) number of framewise outliers; (4) average contrast-to-noise ratio; (5) total susceptibility distortion; and (6) total eddy current-induced distortion (Bastiani et al., 2019; see Quality Control Analyses in *Supplementary Results*).

### Prediction analysis

Partial Least Squares Regression (PLSR) is a data-driven technique designed to reduce high-dimensional datasets into a set of orthogonal components (e.g., factors) that best predict a given outcome or set of outcomes. *Prediction* is operationalized as a model-based approach to predicting an outcome or set of outcomes in a subset of unseen test data from parameters generated within a larger training dataset using cross-validation(Gabrieli et al., 2015; Rudolph et al., 2017, 2018) as described further below. In these analyses, we used PLSR to maximize the covariance between a set of predictors (*x*; the structural connectome) and (*y*; age, fluid cognition, and global WMH) via singular-value decomposition (Abdi & Williams, 2013; Krishnan et al., 2011). *X* is an *n* x *m* matrix where *n* is the set of observations (i.e., participants) and *m* is the full set of unique structural connections. The *X* term thus represents a high-dimensional matrix of correlated data that is to be decomposed with respect to one or more primary variables of interest *(Y).Y* is an *n* x *p* matrix where *p* is each variable of interest (i.e., age, fluid cognition, global WMH). With PLSR, no causality is implied. Partial Least Squares Regression thus provides an unbiased means to assess the relationship among the complex patterns of an individual’s structural connectome and age, fluid cognition, and global WMH, in a single unified model without substantial loss of information or need to impose directionality (e.g., (*X ⇔ Y)* (McIntosh & Lobaugh, 2004). Crucially, by including multiple outcome variables, it is thus possible to assess the relationship between the structural connectome and a target outcome (e.g., global WMH) while considering additional variables of interest (e.g., age and fluid cognition), and, to decompose predictions for each outcome independently. Partial Least Squares Regression models were assessed at both WMH classification thresholds (e.g., *k* = 0.5 and *k* = 0.7).

### Whole-brain prediction

The optimal number of orthogonal components from PLSR models were selected via fivefold cross-validation. Therefore, we split our dataset into five independent sets, constructed prediction models from the training data (four independent sets), and subsequently tested the models on the held-out set of observations (the test set). This process was repeated five times, with each set held out once. The optimal number of components was chosen as that which maximally explained the covariance between age, global WMH, and fluid cognition (cross-validated percent of variance explained; CV-PCTVAR) while minimizing the cross-validated mean-squared error of prediction (CV-MSEP) across each fold. Baseline mean-squared error prior to model fitting is provided for comparison (baseline MSE). Partial Least Squares Regression analyses were conducted in Matlab (The MathWorks, Inc., Natick, MA) using the *plsregress* function with the number of folds set to (*k*) = 5. The importance of a given connection was represented by its beta-weight, which signifies the average contribution of a connection in predicting the covariance between age, fluid cognition, and global WMH (Krishnan et al., 2011; Rudolph et al., 2018) obtained via cross-validation. The unique variance explained for a given outcome is defined by separately comparing the actual or true (y) vs. predicted (y-hat) values for age, fluid cognition, and global WMH. To assess the overall importance of an ROI, the beta-weights for each connection were summed by their absolute value (Rudolph et al., 2017, 2018).

#### Subnetwork prediction

Partial Least Squares Regression models were next generated by restricting connections to within the SUB network (i.e., between pairs of subcortical regions) or between the SUB network and regions falling into one of seven cortical networks (Yeo et al., 2011). Random resampling was used to assess the relative significance of each model compared with random chance (Feczko et al., 2018; Rudolph et al., 2018). Specifically, PLSR models were constructed using an 80/20 train/test split over 5000 iterations to generate a distribution of model performance. On each of the 5000 iterations, the sample was randomly divided into a training set (80%) and a test set (20%) to build and evaluate models respectively. For each randomly selected data set, fivefold cross-validation was applied to determine an unbiased number of optimal components. In addition, on each iteration, the set of target variables (age, fluid cognition, global WMH) were permuted to generate a random null distribution of model performance. Effect size estimates of model significance were obtained by comparing the degree of overlap between the true and empirically generated random null distributions using the Kolmogorov–Smirnov (KS) and Cohen’s *d* statistics. As others have noted, *p*-values are not an appropriate estimate of model significance in this context as *p*-values decrease monotonically with the number of permutations (Combrisson & Jerbi, 2015; Rudolph et al., 2018). In addition to the resampling-based estimates of effect size, we report (1) the median percent of variance explained with their associated confidence intervals, and (2) the average linear association between the observed and predicted values of age, fluid cognition, and global WMH. The latter values were estimated using Pearson’s correlation coefficient (*r*).

### Network analysis

To complement the high-dimensional PLSR analyses, we also tested for linear associations between each of the structural graph metrics of interest (global density, global clustering coefficient, modularity, and global communicability) and age, fluid cognition, and global WMH, using Pearson’s correlations, FDR-corrected for multiple comparisons (Benjamini & Hochberg, 1995).

### Mediation analysis

We conducted mediation analyses to assess whether global and regional WMHs mediated the relationship between age and fluid cognition. Specifically, we were interested in assessing whether age and WMH load had relatively independent influences on fluid cognition within the present data set. Statistical mediation was performed in *R* (R Core Team, 2019) according to the PROCESS framework (Hayes & Rockwood, 2017) using the *bruceR* package (Bao, 2021). Parameter estimates and 95% bootstrapped confidence intervals were obtained using 10,000 bootstrapped samples. Mediation effects were deemed significant if the obtained 95% confidence intervals for a given model excluded zero (Hayes & Rockwood, 2017; Madden et al., 2020, 2014a). In each mediation model, age was considered the predictor and fluid cognition was the outcome. One model included whole-brain WMH as a mediator. A second model included four canonical WM tracts as parallel mediators: the uncinate, forceps major, cingulate bundle (CING1), and the anterior thalamic radiations (ATR). These tracts contain the majority of connections identified in the PLSR models as important for predicting the joint relationship between age, global WMH, and fluid cognition. Because multiple mediators are covaried for each other, this latter model tests whether one or more regions have a mediating effect that is independent of the other regions.

### Post-hoc cognitive domain and test-specific sensitivity analyses

As discussed in the introduction, WMHs have been associated with domain-specific cognitive functions across different levels of analysis. The 12-test neurocognitive battery that we used to construct the latent construct of fluid cognition (NIH Toolbox) is comprised of tests that equally represent the domains of executive function, perceptual speed, and memory. While these three specific cognitive domains are correlated in our data set (*r* = 0.846, 0.604, and 0.557) between executive function and perceptual speed, executive function and memory, and perceptual speed and memory respectively), investigating potential differences across the different tests comprising the neurocognitive battery (NIH Toolbox) battery may be informative. Thus, in supplementary analyses (Figs. S2 and S3 in *Supplementary Material*), linear regression and PLSR models as described above examined relationships between age, cognition, and WMHs using performance on (1) the Dimensional Change Card Sorting Test (indexing executive function), (2) the Pattern Comparison Test speed score (indexing perceptual speed), and (3) the Picture Sequence Memory Test (indexing memory) from the NIH toolbox (see *Methods*) in three separate sets of analyses.

## Results

### Linear relationships between age, fluid cognition, and WMHs

**Fig. 1:**
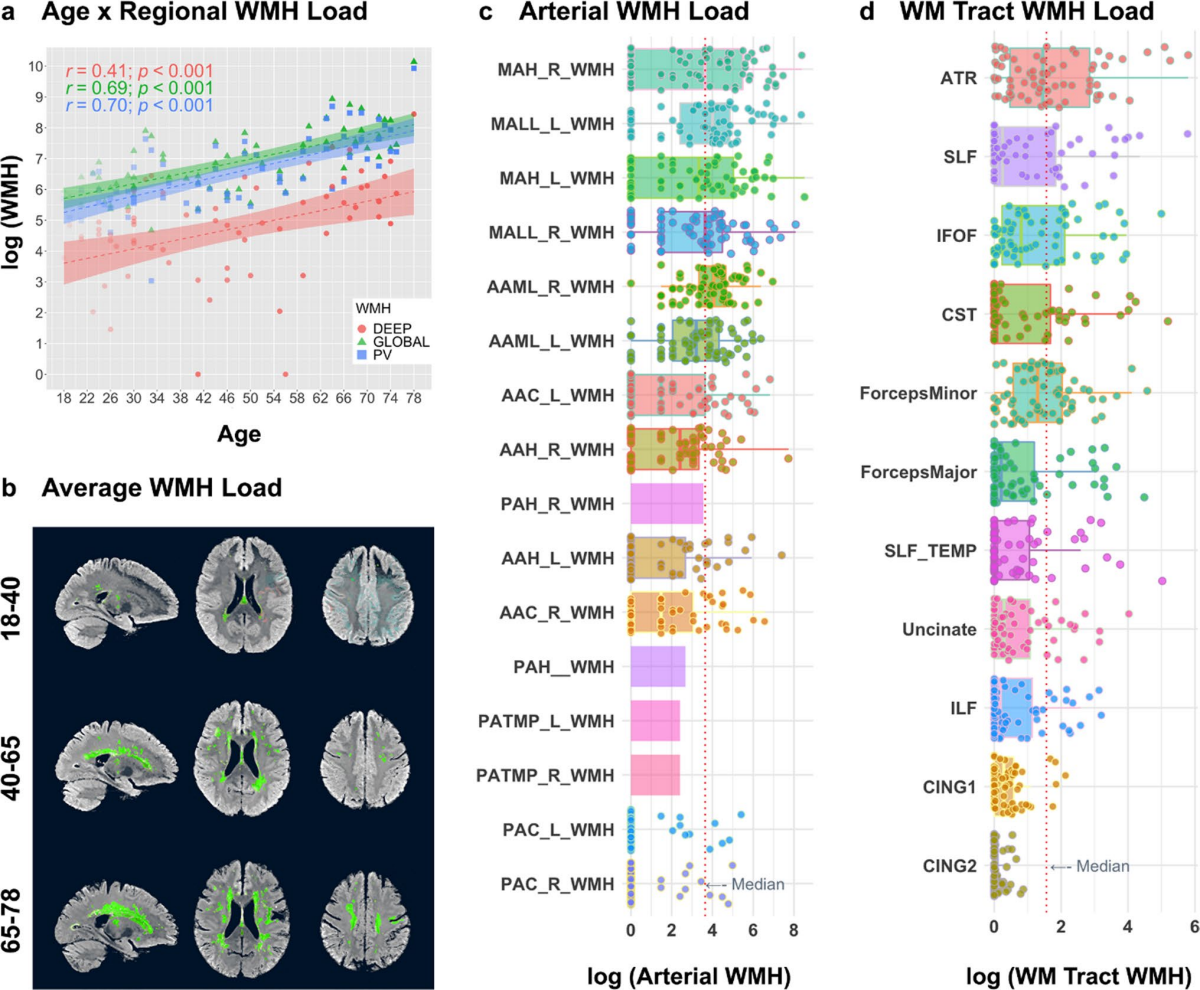
**Global, regional, arterial, and white matter tract-based WMH quantification. a**) There were significant linear associations between age and global WMH (green; triangles), pvWMH (blue; squares), and dWMH (red; circles). **b**) Average WMH maps are shown for the sample of 68 individuals (stratified by age for visualization purposes only). **c**) Log-transformed WMH load for arterial WMH. d) Log-transformed WMH load for white matter tracts. Results are presented using the default classification threshold (*k* = .7; see Table 2; see Supplementary Results and Table S1 for arterial and tract-based results). L = left; R = right; Arterial regions: AAC = anterior artery callosal; AAH = anterior artery hemisphere; MAH = middle artery hemisphere; AAML = anterior artery medial lenticulostriate; MALL = middle artery—lateral lenticulostriate; PATMP = posterior artery thalamic and midbrain perforators; PAH = posterior artery hemisphere; PAC = posterior artery callosal. White Matter Tracts: ATR = anterior thalamic radiations; SLF = superior longitudinal fasciculus; IFOF = inferior frontal-occipital fasciculus; CST = corticospinal tract; ILF = inferior longitudinal fasciculus; CING1 = cingulate bundle 1; CING2 = cingulate bundle 2; Uncinate, uncinate fasciculus

Age was significantly positively associated with global WMH, pvWMH, and dWMH load (*r* = 0.42–0.69, all *p*-FDR ≤ 0.001; Fig. 1a; Table 2). Average WMH probability maps (*k* = 0.7) are shown in Fig. 1b. Age was significantly negatively associated with fluid cognition (*r* =  − 0.859, *p* < 0.0001). Fluid cognition was significantly negatively associated with global WMH, pvWMH, and dWMH load (*r* =  − 0.47 to 0.68, all *p*-FDR ≤ 0.001). Global WMH, pvWMH, and dWMH were significantly correlated with one another (*r* = 0.641–0.998, all *p-*FDR < 0.001). When including age as a covariate in a partial correlation between global WMH and fluid cognition, the relationship between global WMH and fluid cognition remained significant at the default threshold of k = 0.7, but not at a threshold of k = 0.5 (*k* = 0.7: *r* =  − 0.273, *p* = 0.025; *k* = 0.5: *r* =  − 0.071, *p* = 0.563). Additionally, significant associations were found between age, fluid cognition, and both arterial and tract-based WMHs (Table S1).

**Table 2:** Regional WMH load and correlations with age and fluid cognition

	*k* = *.5*							*k* = *.7*						
Regional	*Total*	*M*	*SD*	*r*_*age*_	*p*_-FDR_	*r*_*cog*_	*p*_-FDR_	*Total*	*M*	*SD*	*r*_*age*_	*p*_-FDR_	*r*_*cog*_	*p*_-FDR_
**Global** **WMH**	127,000	6.82	1.03	0.69	< .001	− 0.62	< .001	97,500	6.11	1.40	0.68	< .001	− 0.68	< .001
**pvWMH**	100,000	6.49	1.13	0.69	< .001	− 0.62	< .001	79,100	5.89	1.39	0.65	< .001	− 0.67	< .001
**dWMH**	22,300	4.71	1.69	0.42	.001	− 0.47	.001	16,900	3.52	2.30	0.62	< .001	− 0.62	.001

Summary statistics highlight WMH load and linear associations between age, fluid cognition, and WMHs. Global and pvWMHs were more strongly positively associated with age and negatively associated with fluid cognition than dWMHs. *Total* represents the sum of raw values before log transformation. Values are sorted by total load (*k* = .7). All log-transformed WMH volumes (mm^3^) were significantly associated with age. M = mean; SD = standard deviation; cog = fluid cognition; FDR = false discovery rate

In terms of WMH distribution, arterial WMH load was maximal in the bilateral middle cerebral artery and associated lenticulostriate branches, followed by the medial lenticulostriate branches of the anterior artery (Fig. 1c; Fig. S1a). White matter tract WMH load was maximal in the anterior thalamic radiations (ATR), followed by the inferior fronto-occipital fasciculus (IFOF), superior longitudinal fasciculus (SLF), and corticospinal tract (CST) (Fig. 1d; Fig. S1b; see *Supplementary Results* for additional details).

Finally, a set of analyses were conducted to ensure that results were not due to head motion or quality of the data. No significant associations were found between DWI QC metrics and age or fluid cognition (Table S2) or WMH load (Table S3).

### Prediction analyses

#### Whole-brain prediction

**Fig. 2:**
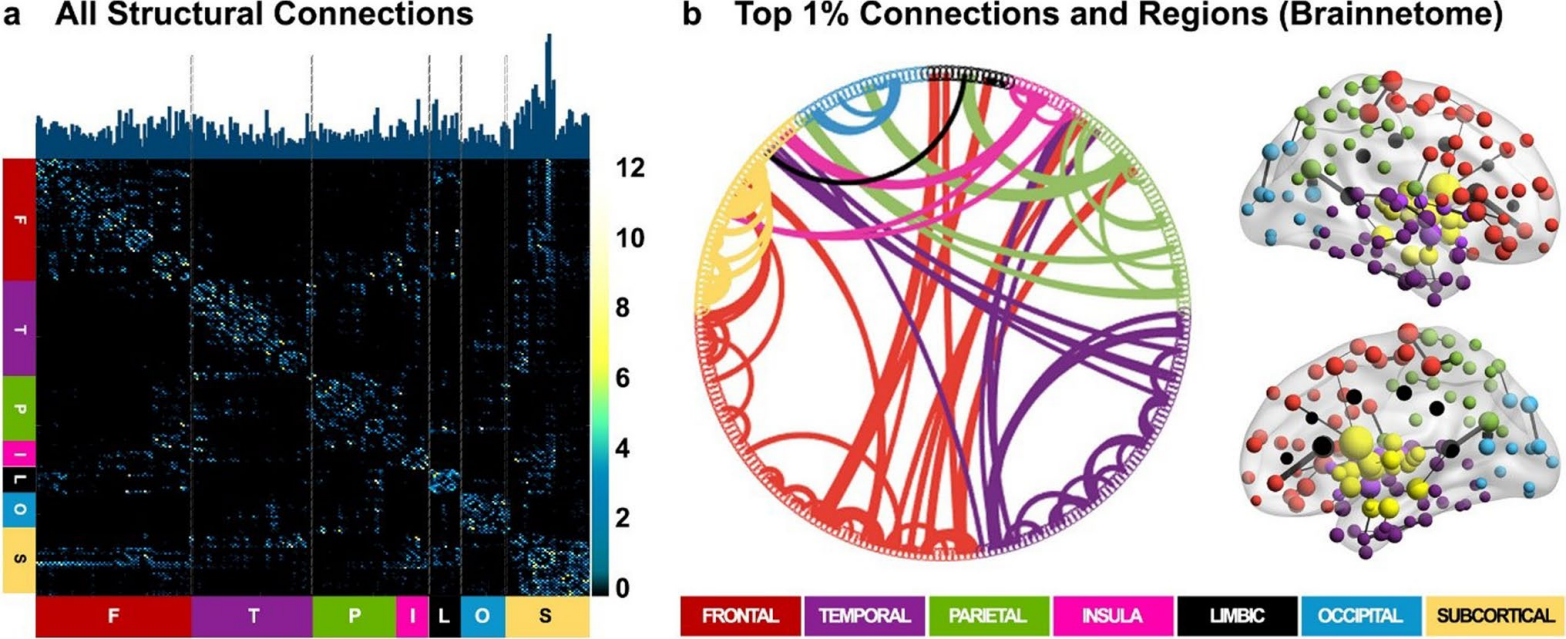
Predicting age, fluid cognition, and global WMH from the whole-brain structural connectome. Predictive features from the whole-brain PLSR model are presented. **a**) Prediction weights (e.g., absolute beta weights) for all structural connections are shown in matrix form (symmetrical matrix; 246 × 246 regions). Each cell represents a connection between a pair of ROIs. Brighter colors represent connections weighted more heavily in the prediction model. The overall importance of an ROI (sum of prediction weights across a row in the matrix) is shown in the bar plot at the top of the matrix. Colored bars below the matrix represent anatomical labels provided by the Brainnetome atlas (F = Frontal (red); T = Temporal (purple); P = Parietal (green); I = Insular (pink); L = Limbic (black); O = Occipital (blue); S = Subcortical (yellow). **b**) The top 1% of connections are plotted for visualization purposes. Connections are displayed as edges between regions arranged by lobe according to the anatomical labels provided by the Brainnetome atlas (**b, left**). The same connections are displayed on a standard brain surface (**b, right**). Lines represent connections between brain regions and are scaled by their prediction weights. ROIs are depicted as spheres and are scaled by the sum of their absolute beta weights. Connections and ROIs are colored according to the Brainnetome atlas labels

Fivefold cross-validated PLSR using all available structural connections achieved an average model accuracy of approximately 99%. Five components maximized the amount of variance explained (*k* = 0.7: CV-PCTVAR = 98.15; *k* = 0.5: CV-PCTVAR = 98.30) and minimized the mean-squared prediction error in the held-out test set across folds (*k* = 0.7: CV-MSEP = 125.49; baseline MSE = 328.26; *k* = 0.5: CV-MSEP = 124.91; baseline MSE = 321.390). All connections contributed to model performance (Fig. 2a). The top connections that predicted the joint association between age, WMH, and fluid cognition were distributed across each of the major lobes, although they were most prominent between pairs of subcortical regions and between subcortical regions and the frontal and parietal lobes (Fig. 2b). To aid with interpretation, we summarized the importance of features at the ROI level. The top-ranked regions primarily consisted of subcortical regions, including the basal ganglia (e.g., bilateral caudate and putamen) and the thalamus (rostro-temporal and caudal-temporal), as well as the cingulate gyrus (Fig. 2; Table 3). Table 3 contains feature importance weights (sum of absolute beta weights) summarized at the level of individual regions for the top 10% (n = 25) ROIs. Prediction weights (e.g., absolute beta weights for each connection) were identical regardless of classification threshold (*r* = 1.0, when comparing models with WMH load estimated at *k* = 0.7 or *k* = 0.5), and when assessing the unique variance explained for each outcome variable (age, global WMH, and fluid cognition) separately. Specifically, prediction weights were near-perfectly correlated when comparing age with WMH (*r* = 0.9987), age with fluid cognition (*r* =  − 0.9998), and WMH with fluid cognition (*r* =  − 0.9977) respectively. Model-derived *r*-squared values for age, WMH, and fluid cognition were 0.993, 0.699, and 0.862 respectively.

**Table 3:** Feature weights

Weight	Lobe	Brainnetome region	Hemisphere
27.32	Subcortical	dCa, dorsal caudate	Right
25.44	Subcortical	dCa, dorsal caudate	Left
17.14	Subcortical	dlPu, dorsolateral putamen	Right
16.20	Subcortical	GP, globus pallidus	Right
15.22	Subcortical	GP, globus pallidus	Left
15.16	Subcortical	dlPu, dorsolateral putamen	Left
13.42	Subcortical	NAC, nucleus accumbens	Left
12.95	Insular	vId/vIg, ventral dysgranular and granular insula	Right
12.90	Limbic	A24rv, rostroventral area 24	Right
12.75	Subcortical	cHipp, caudal hippocampus	Right
12.52	Subcortical	vmPu, ventromedial putamen	Left
12.44	Subcortical	vCa, ventral caudate	Left
11.96	Subcortical	NAC, nucleus accumbens	Right
11.94	Limbic	A24rv, rostroventral area 24	Left
11.42	Frontal	A4t, area 4(trunk region)	Right
11.31	Subcortical	rHipp, rostral hippocampus	Right
11.12	Parietal	dmPOS, dorsomedial parietooccipital sulcus(PEr)	Right
10.96	Subcortical	vmPu, ventromedial putamen	Right
10.88	Frontal	A12/47o, orbital area 12/47	Left
10.71	Subcortical	rTtha, rostral temporal thalamus	Left
10.69	Frontal	A1/2/3ll, area1/2/3 (lower limb region)	Right
10.64	Temporal	A20rv, rostroventral area 20	Left
10.51	Frontal	A44op, opercular area 44	Right
10.29	Subcortical	rTtha, rostral temporal thalamus	Right
10.27	Frontal	A6cvl, caudal ventrolateral area 6	Left

Feature weights for the top 10% of ROIs. ROIs are ranked by the sum of their feature weights

#### Subnetwork prediction

In addition to the whole-brain prediction model, we compared the relative significance of subcortical-subcortical and subcortical-cortical models to a random null distribution using random resampling. All models performed significantly better than chance (all fdr-corrected KS *p*-values < 0.001; Minimum Cohen’s *d* = 1.15). Of the network models assessed, model performance was most variable in the subcortical-to-dorsal attention and subcortical-to-visual networks with lower bounds below zero (Table 4).

**Table 4:** Subnetwork prediction results comparing subcortical-subcortical and subcortical-cortical prediction models to random chance

a. Subcortical- Subcortical	*k* = .5					*k* = .7				
	**KS**	***d***	**Med**	**PI-L**	**PI-H**	**KS**	***d***	**Med**	**PI-L**	**PI-H**
**SUB-SUB**	0.90	2.41	0.58	0.18	0.80	0.90	2.46	0.59	0.19	0.80
**b. Subcortical-** **Cortical**	**KS**	***d***	**Med**	**PI-L**	**PI-H**	**KS**	***d***	**Med**	**PI-L**	**PI-H**
**SUB-DMN**	0.94	2.79	0.51	0.10	0.76	0.94	2.79	0.51	0.08	0.76
**SUB-FPN**	0.90	2.42	0.62	0.28	0.80	0.91	2.52	0.62	0.28	0.80
**SUB-VAN**	0.87	2.21	0.50	0.10	0.75	0.86	2.19	0.50	0.11	0.75
**SUB-DAN**	0.84	2.07	0.49	− 0.03	0.75	0.83	2.02	0.48	− 0.04	0.76
**SUB-LIMBIC**	0.83	2.05	0.60	0.14	0.81	0.83	1.99	0.60	0.15	0.81
**SUB-SMN**	0.74	1.64	0.57	0.11	0.81	0.75	1.63	0.56	0.11	0.80
**SUB-VIS**	0.62	1.15	0.35	− 0.17	0.70	0.62	1.18	0.36	− 0.17	0.70

*Note.* Effect size estimates and prediction statistics are presented for the PLSR models using (a) structural connections between subcortical regions (subcortical-subcortical), or (b) connections between subcortical regions and the 7 cortical networks as defined by Yeo & colleagues (2011). Results are sorted by the Kolmogorov–Smirnov (KS) and Cohen’s d (c) statistics. KS = Kolmogorov–Smirnov; d = Cohen’s d; Med = median; PI-L = Lower bound of 95% prediction interval; PI-H = upper bound of 95% prediction interval; SUB = subcortical network; DMN = default mode network; SMN = somatomotor network; DAN = dorsal attention network; FP = frontoparietal network; LIM = limbic network; VAN = ventral attention network; VIS = visual network

**Fig. 3:**
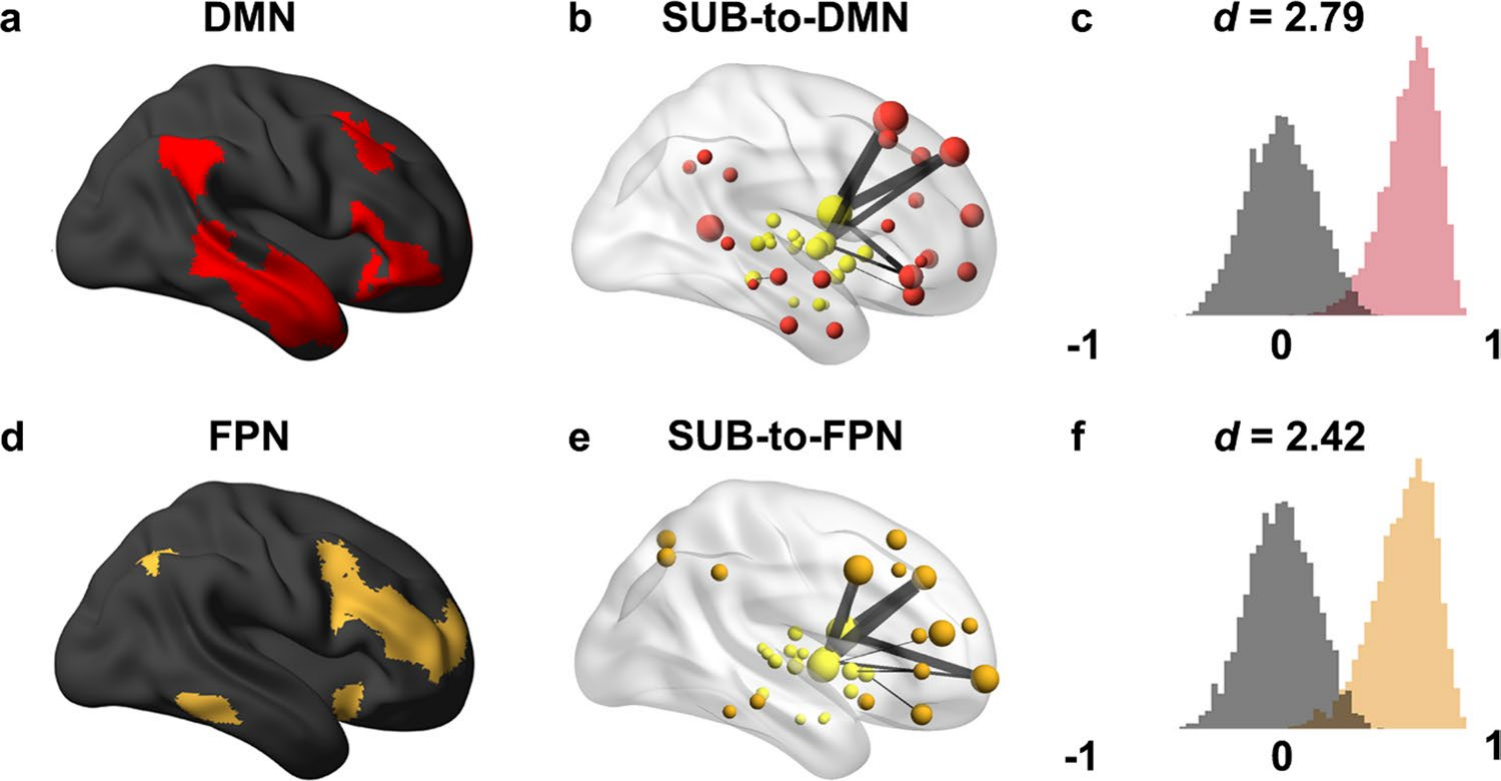
**Subnetwork prediction results comparing top-ranked subcortical-cortical prediction models to chance.** Random resampling was used to assess the relative significance of subcortical-default mode network and subcortical-frontoparietal network models compared to a random null distribution (see Table 4 for results of other network pairs). **a**) Map of the default mode and frontoparietal networks are presented on a standard brain surface. **b**) Top 1% of subcortical-cortical network connections and their corresponding regions are depicted for visualization purposes. Lines represent connections between brain regions and are scaled by their prediction weights. ROIs are depicted as spheres and are scaled by the sum of their absolute beta weights. ROIs are colored by the cortical network. **c**) Depiction of model performance. For a given model, distributions represent the degree of correlation between the actual and predicted outcomes (covariation of age, fluid intelligence, and WMH load) in either the true (colored by network) or random (gray) models over 5000 iterations of the random resampling procedure. Cohen’s *d* effect size measures the distance between the true and random distributions. SUB = subcortical network (yellow); DMN = Default mode network (red); FPN = frontoparietal network (orange)

Top-ranked models included connections among pairs of subcortical regions (SUB-to-SUB; Cohen’s *d* = 2.41) and between subcortical regions and cortical networks containing frontal and parietal regions (Fig. 3), specifically subcortical-to-default mode network (SUB-to-DMN; Cohen’s *d* = 2.79; Figs. 3a–c) and subcortical-to-frontoparietal network (SUB-to-FPN; Cohen’s *d* = 2.42; Figs. 3d–f). This was consistent across WMH classification thresholds (default WMH classification threshold *k* = 0.7 reported above). Findings were not specific to the choice of functional or anatomical parcellation (see *Supplementary Materials*; Table S4).

### Network analyses

Age was negatively associated with global density (*p-*FDR < 0.001) and global communicability (*p-*FDR = 0.002), and positively associated with global clustering coefficient (*p-*FDR = 0.025) and modularity (*p-*FDR =  < 0.001). Fluid cognition was positively associated with global density and global communicability, and negatively associated with modularity (all *p-*FDR < 0.001). As with age, WMHs were also negatively associated with global density (*p-*FDR < 0.001) and global communicability (*p-*FDR = 0.009), and positively associated with modularity (*p-*FDR < 0.001). Fluid cognition and global WMHs were not significantly associated with global clustering coefficient (Table 5). Significant associations were observed between age, fluid cognition, and WMHs with modularity for modules defined both using a data-driven consensus clustering approach and when defined a priori using the Yeo-7 functional network parcellation (all *p-*FDR < 0.001) (Table 5).

**Table 5:** Whole-brain graph theoretical measures and their relationship with age, fluid cognition, and global WMH load

							***Global WMH***			
			**Age**		**Fluid cognition**		***k* = .5**		***k* = .7**	
**Measure**	***M***	***SD***	***r***	***p***_***-FDR***_	***R***	***p***_***-FDR***_	***R***	***p***_***-FDR***_	***r***	***p***_***-FDR***_
**Global density**	0.33	(0.08)	− 0.56	** < .0001**	0.44	** < .000**	− 0.40	**.001**	− 0.38	**.002**
**Global clustering coefficient**	0.01	(0.00)	0.28	**.025**	− 0.19	**.125**	0.11	**0.367**	0.12	0.328
**Global modularity**	0.45	(0.02)	0.57	** < .0001**	− 0.53	** < .0001**	0.49	** < .0001**	0.49	** < .0001**
**Consensus modularity**										
** Gamma = 1**	0.60	(0.02)	0.61	** < 0.001**	− 0.52	** < .0001**	0.50	** < .0001**	0.50	** < .001**
** Gamma = 1.25**	0.56	(0.02)	0.61	**.0001**	− 0.53	** < .0001**	0.52	** < .0001**	0.52	** < .0001**
** Gamma = 1.5**	0.53	(0.02)	0.58	** < .0001**	− 0.61	** < .0001**	0.61	** < .0001**	0.60	** < .0001**
**Global Communicability**	0.18	(0.10)	− 0.36	**.002**	0.31	**.010**	− 0.32	**.009**	− 0.31	**.011**

*Note.* Correlations between graph theoretical metrics of interest and age, fluid cognition, and global WMH load. Modularity was estimated using 8 a priori modules (7 cortical and 1 subcortical). Consensus modularity was estimated using data-driven module assignments at different values of gamma (see *Methods*). *k* = WMH classification threshold; M = mean; SD = standard deviation; FDR = false discovery rate

No models associating global WMH with whole-brain graph theoretical measures survived correction for age and fluid cognition (*p* > 0.05; Table 6). Importantly, age is a defining characteristic of our sample; alterations in structural white matter network organization and WMH load are greater in older adults. Thus, we conducted additional exploratory follow-up analyses assessing relationships between WMH load and structural brain network organization in a subset of middle and older-aged participants (> 45 years), an age range during which WMHs are more prominent. Results using this subset of subjects suggest that higher modularity (e.g., greater segregation) of structural brain networks is moderately associated with greater WMH load in older adults (*r*^2^ = 0.12; *p* = 0.049; see *Supplementary Material*, including Fig. S2).

**Table 6:** Associations between whole-brain graph theoretical measures and global WMH before and after controlling for age and fluid cognition

Model outcome (y)	*Unadjusted*			*Adjusted*		
	*b [95% CI]*	*beta [95% CI]*	*R*^*2*^	*b [95% CI]*	*beta [95% CI]*	*R*^*2*^
**Global density**	− 0.02** [− 0.03, − 0.01]	− 0.38 [− 0.61, − 0.15]	.144**	0 [− 0.02, 0.01]	− 0.06 [− 0.34, 0.22]	.323**
**Global clustering coefficient**	0 [− 0.00, 0.00]	0.12 [− 0.12, 0.37]	.014	0 [− 0.00, 0.00]	− 0.07 [− 0.40, 0.25]	.088**
**Global modularity**	0.01** [0.00, 0.01]	**0.49 [0.28, 0.71]**	.241**	0 [− 0.00, 0.01]	0.2 [− 0.07, 0.48]	.353**
**Consensus modularity**						
** Gamma = 1**	0.01** [0.00, 0.01]	**0.5 [0.29, 0.71]**	.250**	0 [− 0.00, 0.01]	0.2 [− 0.07, 0.46]	.393**
** Gamma = 1.25**	0.01** [0.00, 0.01]	**0.52 [0.31, 0.73]**	.270**	0 [− 0.00, 0.01]	0.22 [− 0.04, 0.48]	.400**
** Gamma = 1.5**	0.01** [0.01, 0.01]	**0.6 [0.40, 0.80]**	.360**	0 [− 0.00, 0.01]	0.28 [0.04, 0.53]	.488**
**Global communicability**	− 0.02** [− 0.04, − 0.01]	** − 0.31 [− 0.55, − 0.08]**	.096**	0 [− 0.03, 0.01]	− 0.14 [− 0.46, 0.17]	.143**

Standard linear regression models assessed associations between global WMH (x) and graph theoretical metrics (y) before (Unadjusted) and after (Adjusted) controlling for age and fluid cognition. *r* represents the zero-order correlation. A significant *b*-weight indicates that the beta-weight and semi-partial correlation are also significant. *b* represents unstandardized regression weights and is equivalent to the unadjusted *r*. *beta* indicates the standardized regression weights. *LL* and *UL* indicate the lower and upper limits of a confidence interval, respectively. *p < .05; **p < .01. Significant coefficients are in bold

### Mediation analysis

Global WMH load significantly mediated the relationship between age and fluid cognition (*ß* =  − 0.006 (0.003), z-value = 2.017, *p* = 0.11; 95% CI = [− 0.013, − 0.001]). However, this was a global rather than regional effect. With the regional WMH values included as parallel mediators, none of them emerged as a significant mediator independent of the others (Table 7).

**Table 7:** Parallel mediation results

Model and variables	Effect	*SE*	z-value	*p*	Lower CI	Upper CI
**Direct effect**						
**Age**	− 0.036	0.005	− 6.626	** < .001**	− 0.047	− 0.026
**Indirect effects**						
Uncinate	− 0.006	0.005	− 1.131	.258	− 0.014	0.005
Forceps major	− 0.004	0.003	− 1.115	.265	− 0.010	0.003
Cingulate bundle 1	− 0.003	0.002	− 1.350	.177	− 0.007	0.001
Anterior thalamic radiations	0.001	0.004	0.230	.818	− 0.008	0.010

### Exploratory post-hoc cognitive domain and test-specific sensitivity analyses

As with fluid cognition, we observed a consistent negative relationship between both age and global WMH with cognitive domain-specific factor scores (executive function: *r* =  − 0.80; perceptual speed: *r* =  − 0.81; memory: *r* =  − 0.64; all *p* < 0.001), as well as with performance on each of the three individual task metrics from the NIH Toolbox (NIH Toolbox Dimensional Change Card Sort Test: *r* =  − 0.66 and *r* =  − 0.58; NIH Toolbox Pattern Comparison Test speed score: *r* =  − 0.66 and *r* =  − 0.36); NIH Toolbox Picture Sequence Memory Test: *r* =  − 0.78 and *r* =  − 0.52; all *p* < 0.001; Fig. S3). Next, we conducted separate predictive models for each of the three individual NIH Toolbox task metrics assessing the joint association between age, WMHs, and cognitive performance (NIH Toolbox Dimensional Change Card Sort Test, NIH Toolbox Pattern Comparison Test speed score, and NIH Toolbox Picture Sequence Memory Test; Fig. S4). These models performed similarly to the models using a single measure of overall fluid cognition with respect to the amount of explained variance in each model and the distributed set of structural connections and regions most important for the predictions (see *Supplementary Material* for details).

## Discussion

The current study combined predictive modeling and network-based analyses to assess how WMHs are associated with age, fluid cognition, and distributed white matter connectivity patterns. Across multiple levels of analysis, we consistently observed that the association between WMHs and altered structural connectivity involved distributed connections between subcortical regions and cortical networks critical for fluid cognition.

Consistent with prior work, WMHs were greater in older compared to younger adults and exhibited a strong negative association with fluid cognition (Bennett & Madden, 2014; Madden et al., 2017; Murray et al., 2010; Raz et al., 2012). Extending prior work, we next showed that WMH load was associated with widespread differences in structural connectivity and network topology. Specifically, whole-brain predictive modeling accurately estimated the joint association between whole-brain structural connectivity and age, fluid cognition, and WMH load. Although connections that were the most predictive were widely distributed, subcortical-subcortical and subcortical-cortical connections were most prominent. When focusing on individual connections, those among pairs of subcortical regions and between subcortical regions and regions of the default mode and frontoparietal networks were the most predictive, especially those between the basal ganglia and prefrontal cortex, and between the thalamus and hippocampus with the temporal cortex. Using a whole-brain model that included all structural connections may have been biased toward identifying short-range connections as most predictive given the sparsity of structural connectivity matrices. Thus, we additionally assessed the relative importance of connectivity of the entire subcortical network with itself and with the seven predefined cortical networks (Yeo et al., 2011). As expected, all subnetwork prediction models were significant compared with random null models. Subnetwork prediction models that were most predictive of the joint relationship between age, fluid cognition, and global WMHs involved connections within the subcortical network (SUB-to-SUB) and between the subcortical network and the default mode (SUB-to-DMN) and frontoparietal (SUB-to-FPN) networks (Fig. 3).

Further extending prior work, we showed that WMHs overlapped with all arterial zones (see *Supplementary Results*). As hypothesized, however, WMHs were most abundant in arterial zones demarcating the middle cerebral artery and associated lenticulostriate branches that supply subcortical regions. Likewise, WMHs overlapped with all major white matter tracts represented within the JHU white matter atlas (Horn & Blankenburg, 2016). However, WMH load was greatest in the anterior thalamic radiations and forceps minor, and highly concentrated in the superior and inferior-occipital longitudinal fasciculi, as well as in the cortico-spinal tracts. Each of these tracts has previously been associated with and thus providing a potential explanation for the relationship between WMH load and fluid cognition (Acharya et al., 2019; Biesbroek et al., 2013, 2017; Kievit et al., 2016). Furthermore, the anterior thalamic radiations support cortico-striatal-thalamic circuits and pathways connecting the thalamus with the anterior cingulate and prefrontal cortices (Biesbroek et al., 2013, 2017). These connections encompass parallel and integrative pathways between the cortex and basal ganglia (Draganski et al., 2008), as well as provide support for the hypothesis that WMHs are diffusely associated with structural connectivity and brain network organization underlying fluid cognition.

The predictive analyses provided an account of how WMHs, which are increased in older adults and associated with poorer fluid cognition, relate to patterns of structural connectivity. To assess associations between WMHs and summary estimates indexing structural brain network topology, we next calculated several graph-theoretical metrics. Global WMHs were negatively associated with global density and global communicability and positively associated with modularity (all *p-*FDR < 0.001). The positive association between global WMH and modularity was observed regardless of whether modules were defined within sample or using a priori networks. Previous work has shown that structural brain networks reorganize by becoming increasingly segregated with age, such that connections are constrained to a greater extent by proximity (Coelho et al., 2021; Madden et al., 2020; Wig, 2017). Our findings suggest that WMHs may serve as one mechanism contributing to the age-related segregation of structural brain networks by disrupting subcortical-cortical communication. Furthermore, communicability is a measure of information transfer via direct and indirect pathways and is one of several metrics used to quantify redundancy, a marker of reserve in brain networks (Langella et al., 2021a, 2021b; Langella et al., 2021a, 2021b; Mišić et al., 2015; Sadiq et al., 2021; Tononi et al., 1999). Although modularity was the only metric associated with WMH load in middle and older-aged adults, negative associations between communicability and both age and WMHs may indicate that altered structural connectivity in aging involves a loss of redundancy, and thus reserve, in structural brain networks.

Altered subcortical-cortical connectivity is likely to play a major role in the loss of redundancy, as subcortical thalamic nuclei are key drivers of cortical functional activation patterns and intrinsic neural oscillations that support fluid cognitive abilities (Bickford, 2015; Fan et al., 2005; Sherman & Guillery, 2002; Shine, 2019; Varela, 2014). The basal ganglia (caudate, putamen, globus pallidus), hippocampus, and thalamus possess high levels of interconnectivity and are intricately connected with the cortex (Oldham & Fornito, 2019). At the network level, highly integrated subcortical and cortical regions (i.e., hubs) facilitate efficient whole-brain communication patterns (Collin et al., 2014; Greene et al., 2020; Griffa  et al., 2018; Kim et al., 2016) to support flexible and adaptive network reconfigurations underlying higher-order cognitive functioning (Cole et al., 2013; Hearne et al., 2017). Importantly, this suggests that WMHs are one potential mechanism that may contribute to global disruption of structural brain connectivity and network organization. This idea is consistent with prior literature suggesting that the impact of WMHs may not be constrained to the specific white matter pathways on which they fall (DeCarli et al., 1995; Langen et al., 2018; Liu et al., 2021; Madden et al., 2017; Wen & Sachdev, 2004).

Finally, in mediation analyses (Hayes & Rockwood, 2017) with age as the predictor and fluid cognition as the outcome we found that global, but not regional, WMH load mediated the relationship between age and fluid cognition (Busby et al., 2023) and that greater WMH load was associated with higher global modularity in older, but not younger adults (see *Supplementary Results*). Thus, these analyses suggest that while WMH load does have a specific influence on the age-cognition relationship, this influence occurs at the level of the whole brain rather than at specific tracts. Given our sample size, we were powered to detect moderate-to-large indirect mediation effects often seen in aging studies comprising both younger and older adults. Because blood pressure and other diagnostic measures were not collected in this sample, we cannot address the impact of cardiovascular or other related factors on age-related differences in WMH deposition. Additional research is needed to assess how both the abundance and spatial distribution of WMHs interact with additional risk factors implicated in age-related cognitive decline, particularly as they emerge in middle age. Understanding how underlying vascular pathology and metabolic dysfunction in particular contribute to age and WMH-related disconnection of white matter networks may help better categorize impairment in both general and specific cognitive domains at the level of an individual.

In the present study, we took several steps to ensure the robustness of our findings. Results could not be attributed to individual differences in image quality, motion artifacts, or choice of WMH segmentation threshold. We additionally quantified global, arterial, and tract-based WMH load, to provide a comprehensive account of WMH deposition seen in our healthy aging cohort. Regardless, findings must be interpreted within the context of the study's limitations. First, owing to incomplete MRI coverage, we excluded the cerebellum from analyses. Several subcortical regions, including the basal ganglia and thalamus, form extensive loops with the cerebellum (Leisman et al., 2014). With that said, WMHs are sparse in the brain stem and cerebellar cortices, and thus the direct impact of WMHs on cortical-cerebellar pathways may be limited (Wen & Sachdev, 2004), but this remains to be tested. Second, given the high degree of age-related variability in regional, arterial, and tract-based WMH estimates, it may be advantageous in future work to model the spatial distribution of WMHs at the voxel or cluster level. Likewise, future work can incorporate nodal estimates of network topology to better understand how individual regions that are impacted by WMHs contribute to local and global dysfunction. Second, to assess the generalizability of these data, the use of larger and more representative samples and independent external validation sets would be valuable. Because these data are cross-sectional, converging evidence from longitudinal data also would be helpful, although both longitudinal and cross-sectional designs have limitations (Salthouse & Nesselroade, 2002; Salthouse, 2011a). Third, in supplementary analyses we showed that age and WMHs were negatively associated with each of the domain-specific cognitive factors of executive function, processing speed, and memory, as well as with individual test scores on the NIH Toolbox Dimensional Change Card Sort Test, NIH Toolbox Pattern Comparison Test speed score, and the NIH Toolbox Picture Sequence Memory Test. Factor scores used to derive our measure of fluid cognition and associations between individual cognitive assessments were highly collinear (see *Supplementary Results*). Thus, we were not able to fully disentangle the relationship between WMHs and specific components of cognition. As discussed within the introduction, however, WMHs have been associated with impaired cognitive processing across general domains of attention, executive functioning, and memory in older adults. Likewise, prior work assessing WMH-related structural disconnection and its impact on cognition in older adults also have reported poorer cognitive functioning in the domains of cognitive control and executive functioning (Langen et al., 2018) and memory retrieval (Jaywant et al., 2022).

In summary, we found that WMHs were more prominent in middle and older-aged adults and were associated with compromised connectivity patterns between subcortical and cortical regions and with poorer fluid cognition. To provide a thorough account of how WMHs are related to age, white matter connectivity, network communication capacity, and fluid cognition, we combined regional- and network-level estimates of structural white matter connectivity with graph-theoretical metrics that summarize network topology. Across multiple levels of analysis, and combining hypothesis- and data-driven methodologies, we showed that altered structural connectivity and greater segregation of structural networks that were related to increased WMHs in older adults were centered on subcortical-subcortical and subcortical-cortical connections, particularly of the default mode and frontoparietal networks. Building from this work, future studies can examine how various biomarkers, including WMHs, influence individual trajectories of age-related cognitive decline, as well as identify individuals at a greater risk for developing dementia.

## Authorship contribution statement

**Marc D. Rudolph:** Conceptualization, Methodology, Formal analysis, Visualization, Writing—original draft, review & editing. **Jessica R. Cohen:** Advice, Methods Discussion, Writing—Review & Editing. **David J. Madden:** Funding Acquisition, Supervision, Methods Discussion, Writing—Review & Editing.

## Supplementary information

Below is the link to the electronic supplementary material.

Supplementary file1 (DOCX 3059 KB)

## Data Availability

Data will be made available upon reasonable request, based on approval from the requesting researcher's local ethics committee. Depending on the nature of the request, a formal data-sharing agreement may be required.

## Code availability

Code will be made available upon reasonable request.

## Declarations

### Open Practices Statements

Data or materials for the experiments are available upon request as described above, and none of the experiments were preregistered.

## References

[CR1] Abdi, H., & Williams, L. J. (2013). Partial least squares methods: Partial least squares correlation and partial least square regression. *Methods in Molecular Biology,**930*, 549–579. 10.1007/978-1-62703-059-5_23 23086857 10.1007/978-1-62703-059-5_23

[CR2] Acharya, A., Liang, X., Tian, W., Jiang, C., Han, Y., & Yi, L. (2019). White matter hyperintensities relate to basal ganglia functional connectivity and memory performance in aMCI and SVMCI. *Frontiers in Neuroscience*, 13. 10.3389/fnins.2019.01204 10.3389/fnins.2019.01204 PMC6874172 31798401

[CR3] Alexander, G. E., DeLong, M. R., & Strick, P. L. (1986). Parallel organization of functionally segregated circuits linking basal ganglia and cortex. *Annual Review of Neuroscience*, 357–381. 10.1146/annurev.ne.09.030186.002041 10.1146/annurev.ne.09.030186.002041 3085570

[CR4] Andersson, J. L. R., Skare, S., & Ashburner, J. (2003). How to correct susceptibility distortions in spin-echo echo-planar images: Application to diffusion tensor imaging. *NeuroImage,**20*(2), 870–888. 10.1016/S1053-8119(03)00336-7 14568458 10.1016/S1053-8119(03)00336-7

[CR5] Ashburner, J. (2007). A fast diffeomorphic image registration algorithm. *NeuroImage,**38*(1), 95–113. 10.1016/j.neuroimage.2007.07.007 17761438 10.1016/j.neuroimage.2007.07.007

[CR6] Bach, M. (1996). The Freiburg Visual Acuity Test - automatic measurement of visual acuity. *Optometry and Vision Science,**73*(1), 49–53. 10.1097/00006324-199601000-00008 8867682 10.1097/00006324-199601000-00008

[CR7] Baronchelli, A., Ferrer-i-Cancho, R., Pastor-Satorras, R., Chater, N., & Christiansen, M. H. (2013). *Networks in Cognitive Science,**17*(7), 348–360. 10.1016/j.tics.2013.04.010 10.1016/j.tics.2013.04.010 23726319

[CR8] Bassett, D. S., Porter, M. A., Wymbs, N. F., Grafton, S. T., Carlson, J. M., & Mucha, P. J. (2013). Robust detection of dynamic community structure in networks. *Chaos*, 23(1). 10.1063/1.4790830 10.1063/1.4790830 PMC3618100 23556979

[CR9] Bastiani, M., Cottaar, M., Fitzgibbon, S. P., Suri, S., Alfaro-Almagro, F., Sotiropoulos, S. N., Jbabdi, S., & Andersson, J. L. R. (2019). Automated quality control for within and between studies diffusion MRI data using a non-parametric framework for movement and distortion correction. *NeuroImage,**184*, 801–812. 10.1016/j.neuroimage.2018.09.073 30267859 10.1016/j.neuroimage.2018.09.073 PMC6264528

[CR10] Beck, A. T., Ward, C. H., Mendelson, M., Mock, J., & Erbaugh, J. (1961). An inventory for measuring depression. *Archives of General Psychiatry,**4*(6), 561–571. 10.1001/archpsyc.1961.01710120031004 13688369 10.1001/archpsyc.1961.01710120031004

[CR11] Benjamini, Y., & Hochberg, Y. (1995). Controlling the false discovery rate: A practical and powerful approach to multiple testing. *Journal of the Royal Statistical Society,**57*(1), 289–300. 10.2307/2346101

[CR12] Bennett, I. J., & Madden, D. J. (2014). Disconnected aging: Cerebral white matter integrity and age-related differences in cognition. *Neuroscience,**276*, 187–205. 10.1016/j.neuroscience.2013.11.026 24280637 10.1016/j.neuroscience.2013.11.026 PMC4032380

[CR13] Betzel, R. F., Medaglia, J. D., Papadopoulos, L., Baum, G. L., Gur, R., Gur, R., Roalf, D., Satterthwaite, T. D., & Bassett, D. S. (2017). The modular organization of human anatomical brain networks: Accounting for the cost of wiring. *Network Neuroscience,**1*(1), 42–68. 10.1162/NETN_a_00002 30793069 10.1162/NETN_a_00002 PMC6372290

[CR14] Bickford, M. E. (2015). Thalamic circuit diversity: modulation of the driver/modulator framework. *Frontiers in Neural Circuits*, 9(JAN2016). 10.3389/fncir.2015.00086 10.3389/fncir.2015.00086 PMC4709853 26793068

[CR15] Biesbroek, J. M., Kuijf, H. J., van der Graaf, Y., Vincken, K. L., Postma, A., Mali, W. P. T. M., Biessels, G. J., & Geerlings, M. I. (2013). Association between subcortical vascular lesion location and cognition: a voxel-based and tract-based lesion-symptom mapping study. The SMART-MR Study. *PLoS ONE*, 8(4). 10.1371/journal.pone.0060541 10.1371/journal.pone.0060541 PMC3620525 23593238

[CR16] Biesbroek, J. M., Weaver, N. A., & Biessels, G. J. (2017). Lesion location and cognitive impact of cerebral small vessel disease. *Clinical Science,**131*(8), 715–728. 10.1042/CS20160452 28385827 10.1042/CS20160452

[CR17] Birdsill, A. C., Koscik, R. L., Jonaitis, E. M., Johnson, S. C., Okonkwo, O. C., Hermann, B. P., LaRue, A., Sager, M. A., & Bendlin, B. B. (2014). Regional white matter hyperintensities: Aging, Alzheimer’s disease risk, and cognitive function. *Neurobiology of Aging,**35*(4), 769–776. 10.1016/j.neurobiolaging.2013.10.072 24199958 10.1016/j.neurobiolaging.2013.10.072 PMC3880609

[CR18] Blondel, V. D., Guillaume, J. L., Lambiotte, R., & Lefebvre, E. (2008). Fast unfolding of communities in large networks. *Journal of Statistical Mechanics: Theory and Experiment,**2008*(10), P10008. 10.1088/1742-5468/2008/10/P10008

[CR19] Boot, E. M., MC van Leijsen, E., Bergkamp, M. I., Kessels, R. P. C., Norris, D. G., de Leeuw, F. E., & Tuladhar, A. M. (2020). Structural network efficiency predicts cognitive decline in cerebral small vessel disease. *NeuroImage: Clinical*, 27, 102325. 10.1016/j.nicl.2020.102325 10.1016/j.nicl.2020.102325 PMC7334365 32622317

[CR20] Boots, E. A., Zhan, L., Dion, C., Karstens, A. J., Peven, J. C., Ajilore, O., & Lamar, M. (2019). Cardiovascular disease risk factors, tract-based structural connectomics, and cognition in older adults. *NeuroImage,**196*, 152–160. 10.1016/j.neuroimage.2019.04.024 30980900 10.1016/j.neuroimage.2019.04.024 PMC6713222

[CR21] Bressler, S. L., & Tognoli, E. (2006). Operational principles of neurocognitive networks. *International Journal of Psychophysiology,**60*(2), 139–148. 10.1016/j.ijpsycho.2005.12.008 16490271 10.1016/j.ijpsycho.2005.12.008

[CR22] Brugulat-Serrat, A., Salvadó, G., Operto, G., Cacciaglia, R., Sudre, C. H., Grau-Rivera, O., Suárez-Calvet, M., Falcon, C., Sánchez-Benavides, G., Gramunt, N., Minguillon, C., Fauria, K., Barkhof, F., Molinuevo, J. L., & Gispert, J. D. (2020a). White matter hyperintensities mediate gray matter volume and processing speed relationship in cognitively unimpaired participants. *Human Brain Mapping,**41*(5), 1309–1322. 10.1002/hbm.24877 31778002 10.1002/hbm.24877 PMC7267988

[CR23] Brugulat-Serrat, A., Salvadó, G., Sudre, C. H., Grau-Rivera, O., Suárez-Calvet, M., Falcon, C., Sánchez-Benavides, G., Gramunt, N., Fauria, K., Cardoso, M. J., Barkhof, F., Molinuevo, J. L., & Gispert, J. D. (2020b). Patterns of white matter hyperintensities associated with cognition in middle-aged cognitively healthy individuals. *Brain Imaging and Behavior,**14*(5), 2012–2023. 10.1007/s11682-019-00151-2 31278650 10.1007/s11682-019-00151-2 PMC7572336

[CR24] Busby, N., Wilson, S., Wilmskoetter, J., Newman-Norlund, R., Sayers, S., Newman-Norlund, S.,

[CR25] Roth, R., Rorden, C., Fridriksson, J., & Bonilha, L. (2023). White matter hyperintensity load mediates the relationship between age and cognition. *Neurobiology of Aging,**132*, 56–66. 10.1016/J.NEUROBIOLAGING.2023.08.007 37729770 10.1016/j.neurobiolaging.2023.08.007 PMC12573672

[CR26] Caligiuri, M. E., Perrotta, P., Augimeri, A., Rocca, F., Quattrone, A., & Cherubini, A. (2015). Automatic detection of white matter hyperintensities in healthy aging and pathology using magnetic resonance imaging: A review. *Neuroinformatics,**13*(3), 261–276. 10.1007/s12021-015-9260-y 25649877 10.1007/s12021-015-9260-y PMC4468799

[CR27] Chen, H., Huang, L., Yang, D., Ye, Q., Guo, M., Qin, R., Luo, C., Li, M., Ye, L., Zhang, B., & Xu, Y. (2019). Nodal global efficiency in front-parietal lobe mediated periventricular white matter hyperintensity (PWMH)-related cognitive impairment. *Frontiers in Aging Neuroscience*, 11. 10.3389/fnagi.2019.00347 10.3389/fnagi.2019.00347 PMC6914700 31920627

[CR28] Coelho, A., Fernandes, H. M., Magalhães, R., Moreira, P. S., Marques, P., Soares, J. M., Amorim, L., Portugal-Nunes, C., Castanho, T., Santos, N. C., & Sousa, N. (2021). Reorganization of brain structural networks in aging: A longitudinal study. *Journal of Neuroscience Research,**99*(5), 1354. 10.1002/JNR.24795 33527512 10.1002/jnr.24795 PMC8248023

[CR29] Cole, M. W., Reynolds, J. R., Power, J. D., Repovs, G., Anticevic, A., & Braver, T. S. (2013). Multi-task connectivity reveals flexible hubs for adaptive task control. *Nature Neuroscience,**16*(9), 1348–1355. 10.1038/nn.3470 23892552 10.1038/nn.3470 PMC3758404

[CR30] Collin, G., Sporns, O., Mandl, R. C. W., & Van Den Heuvel, M. P. (2014). Structural and functional aspects relating to cost and benefit of rich club organization in the human cerebral cortex. *Cerebral Cortex,**24*(9), 2258–2267. 10.1093/cercor/bht064 23551922 10.1093/cercor/bht064 PMC4128699

[CR31] Combrisson, E., & Jerbi, K. (2015). Exceeding chance level by chance: The caveat of theoretical chance levels in brain signal classification and statistical assessment of decoding accuracy. *Journal of Neuroscience Methods,**250*, 126–136. 10.1016/j.jneumeth.2015.01.010 25596422 10.1016/j.jneumeth.2015.01.010

[CR32] Craik, F. I. M., & Bialystok, E. (2006). Cognition through the lifespan: Mechanisms of change. *Trends in Cognitive Sciences,**10*(3), 131–138. 10.1016/j.tics.2006.01.007 16460992 10.1016/j.tics.2006.01.007

[CR33] de Groot, J. C., de Leeuw, F. E., Oudkerk, M., Hofman, A., Jolles, J., & Breteler, M. M. B. (2001). Cerebral white matter lesions and subjective cognitive dysfunction: The Rotterdam scan study. *Neurology,**56*(11), 1539–1545. 10.1212/WNL.56.11.1539 11402112 10.1212/wnl.56.11.1539

[CR34] DeCarli, C., Murphy, D. G. M., Tranh, M., Grady, C. L., Haxby, J., & v., Gillette, J. A., Salerno, J. A., Gonzales-Aviles, A., Honvitz, B., Rapoport, S. I., & Schapiro, M. B. (1995). The effect of white matter hyperintensity volume on brain structure, cognitive performance, and cerebral metabolism of glucose in 51 healthy adults. *Neurology,**45*(11), 2077–2084. 10.1212/WNL.45.11.2077 7501162 10.1212/wnl.45.11.2077

[CR35] Delis, D. C., Kramer, J. H., Kaplan, E., & Holdnack, J. (2004). Reliability and validity of the Delis-Kaplan Executive Function System: An update. *Journal of the International Neuropsychological Society,**10*(2), 301–303. 10.1017/S1355617704102191 15012851 10.1017/S1355617704102191

[CR36] Draganski, B., Kherif, F., Klöppel, S., Cook, P. A., Alexander, D. C., Parker, G. J. M., Deichmann, R., Ashburner, J., & Frackowiak, R. S. J. (2008). Evidence for segregated and integrative connectivity patterns in the human basal ganglia. *Journal of Neuroscience,**28*(28), 7143–7152. 10.1523/JNEUROSCI.1486-08.2008 18614684 10.1523/JNEUROSCI.1486-08.2008 PMC6670486

[CR37] Estrada, E., & Hatano, N. (2008). Communicability in complex networks. *Physical Review E - Statistical, Nonlinear, and Soft Matter Physics,**77*(3), 036111. 10.1103/PhysRevE.77.036111 18517465 10.1103/PhysRevE.77.036111

[CR38] Fama, R., & Sullivan, E. V. (2015). Thalamic structures and associated cognitive functions: Relations with age and aging. *Neuroscience and Biobehavioral Reviews,**54*, 29–37. 10.1016/j.neubiorev.2015.03.008 25862940 10.1016/j.neubiorev.2015.03.008 PMC4457546

[CR39] Fan, J., McCandliss, B. D., Fossella, J., Flombaum, J. I., & Posner, M. I. (2005). The activation of attentional networks. *NeuroImage,**26*(2), 471–479. 10.1016/j.neuroimage.2005.02.004 15907304 10.1016/j.neuroimage.2005.02.004

[CR40] Fan, L., Li, H., Zhuo, J., Zhang, Y., Wang, J., Chen, L., Yang, Z., Chu, C., Xie, S., Laird, A. R., Fox, P. T., Eickhoff, S. B., Yu, C., & Jiang, T. (2016). The Human Brainnetome Atlas: A new brain atlas based on connectional architecture. *Cerebral Cortex,**26*(8), 3508–3526. 10.1093/cercor/bhw157 27230218 10.1093/cercor/bhw157 PMC4961028

[CR41] Faul, F., Erdfelder, E., Lang, A. G., & Buchner, A. (2007). G*Power 3: A flexible statistical power analysis program for the social, behavioral, and biomedical sciences. *Behavior Research Methods,**39*, 175–191. 10.3758/BF03193146 17695343 10.3758/bf03193146

[CR42] Feczko, E., Balba, N. M., Miranda-Dominguez, O., Cordova, M., Karalunas, S. L., Irwin, L., Demeter, D. V., Hill, A. P., Langhorst, B. H., Grieser Painter, J., Van Santen, J., Fombonne, E. J., Nigg, J. T., & Fair, D. A. (2018). Subtyping cognitive profiles in autism spectrum disorder using a Functional Random Forest algorithm. *NeuroImage,**172*, 674–688. 10.1016/j.neuroimage.2017.12.044 29274502 10.1016/j.neuroimage.2017.12.044 PMC5969914

[CR43] Folstein, M. F., Folstein, S. E., & McHugh, P. R. (1975). “Mini-mental state”: A practical method for grading the cognitive state of patients for the clinician. *Journal of Psychiatric Research,**12*(3), 189–198. 10.1016/0022-3956(75)90026-6 1202204 10.1016/0022-3956(75)90026-6

[CR44] Frey, B. M., Petersen, M., Mayer, C., Schulz, M., Cheng, B., & Thomalla, G. (2019). Characterization of white matter hyperintensities in large-scale MRI-studies. *Frontiers in Neurology*, 10. 10.3389/fneur.2019.00238 10.3389/fneur.2019.00238 PMC6443932 30972001

[CR45] Gershon, R. C., Wagster, M. V., Hendrie, H. C., Fox, N. A., Cook, K. F., & Nowinski, C. J. (2013). NIH Toolbox for assessment of neurological and behavioral function. *Neurology*, 80(Issue 11, Supplement 3), S2–S6. 10.1212/WNL.0b013e3182872e5f 10.1212/WNL.0b013e3182872e5f PMC3662335 23479538

[CR46] Greene, D. J., Marek, S., Gordon, E. M., Siegel, J. S., Gratton, C., Laumann, T. O., Gilmore, A. W., Berg, J. J., Nguyen, A. L., Dierker, D., Van, A. N., Ortega, M., Newbold, D. J., Hampton, J. M., Nielsen, A. N., McDermott, K. B., Roland, J. L., Norris, S. A., Nelson, S. M., … Dosenbach, N. U. F. (2020). Integrative and network-specific connectivity of the basal ganglia and thalamus defined in individuals. *Neuron,**105*(4), 742-758.e6.10.1016/j.neuron.2019.11.012 31836321 10.1016/j.neuron.2019.11.012 PMC7035165

[CR47] Griffa, A., & Van den Heuvel, M. P. (2018). Rich-club neurocircuitry: Function, evolution, and vulnerability. *Dialogues in Clinical Neuroscience,**20*(2), 121–132. 30250389 10.31887/DCNS.2018.20.2/agriffa PMC6136122

[CR48] Gunning-Dixon, F. M., & Raz, N. (2003). Neuroanatomical correlates of selected executive functions in middle-aged and older adults: A prospective MRI study. *Neuropsychologia,**41*(14), 1929–1941. 10.1016/S0028-3932(03)00129-5 14572526 10.1016/s0028-3932(03)00129-5

[CR49] Hachinski, V. C., Potter, P., & Merskey, H. (1987). Leuko-Araiosis. *Archives of Neurology,**44*(1), 21–23. 10.1001/archneur.1987.00520130013009 3800716 10.1001/archneur.1987.00520130013009

[CR50] Hagmann, P., Cammoun, L., Gigandet, X., Meuli, R., Honey, C. J., Wedeen, V. J., & Sporns, O. (2008). Mapping the structural core of human cerebral cortex. *PLoS Biology,**6*(7), e159. 10.1371/journal.pbio.0060159v 18597554 10.1371/journal.pbio.0060159 PMC2443193

[CR51] Hayes, A. F., & Rockwood, N. J. (2017). Regression-based statistical mediation and moderation analysis in clinical research: Observations, recommendations, and implementation. *Behaviour Research and Therapy,**98*, 39–57. 10.1016/J.BRAT.2016.11.001 27865431 10.1016/j.brat.2016.11.001

[CR52] Hearne, L. J., Cocchi, L., Zalesky, A., & Mattingley, J. B. (2017). Reconfiguration of brain network architectures between resting state and complexity-dependent cognitive reasoning. *Journal of Neuroscience,**37*(35), 0485–0517. 10.1523/JNEUROSCI.0485-17.2017 10.1523/JNEUROSCI.0485-17.2017 PMC6596866 28760864

[CR53] Hedden, T., Mormino, E. C., Amariglio, R. E., Younger, A. P., Schultz, A. P., Becker, J. A., Buckner, R. L., Johnson, K. A., Sperling, R. A., & Rentz, D. M. (2012). Cognitive profile of amyloid burden and white matter hyperintensities in cognitively normal older adults. *Journal of Neuroscience,**32*(46), 16233–16242. 10.1523/JNEUROSCI.2462-12.2012 23152607 10.1523/JNEUROSCI.2462-12.2012 PMC3523110

[CR54] Hedden, T., Schultz, A. P., Rieckmann, A., Mormino, E. C., Johnson, K. A., Sperling, R. A., & Buckner, R. L. (2016). Multiple brain markers are linked to age-related variation in cognition. *Cerebral Cortex,**26*(4), 1388–1400. 10.1093/cercor/bhu238 25316342 10.1093/cercor/bhu238 PMC4785941

[CR55] Hoagey, D. A., Lazarus, L. T. T., Rodrigue, K. M., & Kennedy, K. M. (2021). The effect of vascular health factors on white matter microstructure mediates age-related differences in executive function performance. *Cortex,**141*, 403–420. 10.1016/J.CORTEX.2021.04.016 34130048 10.1016/j.cortex.2021.04.016 PMC8319097

[CR56] Horn, A., & Blankenburg, F. (2016). Toward a standardized structural-functional group connectome in MNI space. *NeuroImage,**124*, 310–322. 10.1016/j.neuroimage.2015.08.048 26327244 10.1016/j.neuroimage.2015.08.048

[CR57] Jaywant, A., Dunlop, K., Victoria, L. W., Oberlin, L., Lynch, C. J., Respino, M., Kuceyeski, A., Scult, M., Hoptman, M. J., Liston, C., O’Dell, M. W., Alexopoulos, G. S., Perlis, R. H., & Gunning, F. M. (2022). Estimated regional white matter hyperintensity burden, resting state functional connectivity, and cognitive functions in older adults. *The American Journal of Geriatric Psychiatry,**30*(3), 269–280. 10.1016/j.jagp.2021.07.015 34412936 10.1016/j.jagp.2021.07.015 PMC8799753

[CR58] Jiang, J., Liu, T., Zhu, W., Koncz, R., Liu, H., Lee, T., Sachdev, P. S., & Wen, W. (2018a). UBO detector – a cluster-based, fully automated pipeline for extracting white matter hyperintensities. *NeuroImage,**174*, 539–549. 10.1016/j.neuroimage.2018.03.050 29578029 10.1016/j.neuroimage.2018.03.050

[CR59] Jiang, J., Paradise, M., Liu, T., Armstrong, N. J., Zhu, W., Kochan, N. A., Brodaty, H., Sachdev, P. S., & Wen, W. (2018). The association of regional white matter lesions with cognition in a community-based cohort of older individuals. *NeuroImage: Clinical*, 19, 14–21. 10.1016/j.nicl.2018.03.035 10.1016/j.nicl.2018.03.035 PMC6051317 30034997

[CR60] Jung, K. H., Stephens, K. A., Yochim, K. M., Riphagen, J. M., Kim, C. M., Buckner, R. L., & Salat, D. H. (2021). Heterogeneity of cerebral white matter lesions and clinical correlates in older adults. *Stroke*, 620–630. 10.1161/STROKEAHA.120.031641 10.1161/STROKEAHA.120.031641 PMC9477514 33406867

[CR61] Kertesz, A., Black, S. E., Tokar, G., Benke, T., Carr, T., & Nicholson, L. (1988). Periventricular and subcortical hyperintensities on magnetic resonance imaging: ‘Rims, caps, and unidentified bright objects.’ *Archives of Neurology,**45*(4), 404–408. 10.1001/archneur.1988.00520280050015 3355395 10.1001/archneur.1988.00520280050015

[CR62] Kievit, R. A., Davis, S. W., Griffiths, J., Correia, M. M., & Cam-CAN, & Henson, R. N. (2016). A watershed model of individual differences in fluid intelligence. *Neuropsychologia,**91*, 186–198. 10.1016/j.neuropsychologia.2016.08.008 27520470 10.1016/j.neuropsychologia.2016.08.008 PMC5081064

[CR63] Kim, D. J., Davis, E. P., Sandman, C. A., Sporns, O., O’Donnell, B. F., Buss, C., & Hetrick, W. P. (2016). Children’s intellectual ability is associated with structural network integrity. *NeuroImage,**124*(Pt A), 550–556. 10.1016/j.neuroimage.2015.09.012 26385010 10.1016/j.neuroimage.2015.09.012 PMC4651770

[CR64] Kim, K. W., MacFall, J. R., & Payne, M. E. (2008). Classification of white matter lesions on magnetic resonance imaging in elderly persons. *Biological Psychiatry,**64*(4), 273–280. 10.1016/j.biopsych.2008.03.024 18471801 10.1016/j.biopsych.2008.03.024 PMC2593803

[CR65] Krishnan, A., Williams, L. J., McIntosh, A. R., & Abdi, H. (2011). Partial Least Squares (PLS) methods for neuroimaging: A tutorial and review. *NeuroImage,**56*(2), 455–475. 10.1016/j.neuroimage.2010.07.034 20656037 10.1016/j.neuroimage.2010.07.034

[CR66] Lancichinetti, A., & Fortunato, S. (2012). Consensus clustering in complex networks. *Scientific Reports*, 2. 10.1038/SREP00336 10.1038/srep00336 PMC3313482 22468223

[CR67] Langella, S., Mucha, P. J., Giovanello, K. S., & Dayan, E. (2021a). The association between hippocampal volume and memory in pathological aging is mediated by functional redundancy. *Neurobiology of Aging,**108*, 179–188. 10.1016/J.NEUROBIOLAGING.2021.09.002 34614422 10.1016/j.neurobiolaging.2021.09.002 PMC8616845

[CR68] Langella, S., Sadiq, M. U., Mucha, P. J., Giovanello, K. S., & Dayan, E. (2021b). Lower functional hippocampal redundancy in mild cognitive impairment. *Translational Psychiatry,**11*(1), 1–12. 10.1038/s41398-020-01166-w 33462184 10.1038/s41398-020-01166-w PMC7813821

[CR69] Langen, C. D., Cremers, L. G. M., de Groot, M., White, T., Ikram, M. A., Niessen, W. J., & Vernooij, M. W. (2018). Disconnection due to white matter hyperintensities is associated with lower cognitive scores. *NeuroImage,**183*, 745–756. 10.1016/j.neuroimage.2018.08.037 30144572 10.1016/j.neuroimage.2018.08.037

[CR70] Langen, C. D., Vernooij, M. W., Cremers, L. G. M., Huizinga, W., De Groot, M., Ikram, M. A., White, T., & Niessen, W. J. (2017). The structural disconnectome: A pathology-sensitive extension of the structural connectome, 2017 IEEE 14th International Symposium on Biomedical Imaging (ISBI 2017). *Melbourne, VIC, Australia,**2017*, 366–370. 10.1109/ISBI.2017.7950539

[CR71] Leisman, G., Braun-Benjamin, O., & Melillo, R. (2014). Cognitive-motor interactions of the basal ganglia in development. *Frontiers in Systems Neuroscience,**8*, 16. 10.3389/fnsys.2014.00016 24592214 10.3389/fnsys.2014.00016 PMC3923298

[CR72] Li, Q., Yang, Y., Reis, C., Tao, T., Li, W., Li, X., & Zhang, J. H. (2018). Cerebral Small Vessel Disease. *Cell Transplantation,**27*(12), 1711–1722. 10.1177/0963689718795148 30251566 10.1177/0963689718795148 PMC6300773

[CR73] Li, Z., Dolui, S., Habes, M., Bassett, D. S., Wolk, D., & Detre, J. A. (2021). Predicted disconnectome associated with progressive periventricular white matter ischemia. *Cerebral Circulation - Cognition and Behavior,* 2. 10.1016/j.cccb.2021.100022 10.1016/j.cccb.2021.100022 PMC9616229 36324715

[CR74] Liu, Y., Xia, Y., Wang, X., Wang, Y., Zhang, D., Nguchu, B. A., He, J., Wang, Y., Yang, L., Wang, Y., Ying, Y., Liang, X., Zhao, Q., Wu, J., Liang, Z., Ding, D., Dong, Q., Qiu, B., Cheng, X., & Gao, J. H. (2021). White matter hyperintensities induce distal deficits in the connected fibers. *Human Brain Mapping,**42*(6), 1910–1919. 10.1002/hbm.25338 33417309 10.1002/hbm.25338 PMC7978134

[CR75] Li, Y., Kalpouzos, G., Bäckman, L., Qiu, C., & Laukka, E. J. (2023). Association of white matter hyperintensity accumulation with domain-specific cognitive decline: a population-based cohort study. *Neurobiology of Aging. * 10.1016/J.NEUROBIOLAGING.2023.08.011 10.1016/j.neurobiolaging.2023.08.011 37776581

[CR76] Lockhart, S. N., Luck, S. J., Geng, J., Beckett, L., Disbrow, E. A., Carmichael, O., & DeCarli, C. (2015). White matter hyperintensities among older adults are associated with futile increase in frontal activation and functional connectivity during spatial search. *PLoS ONE*, 10(3). 10.1371/journal.pone.0122445 10.1371/journal.pone.0122445 PMC4368687 25793922

[CR77] Lockhart, S. N., Roach, A. E., Luck, S. J., Geng, J., Beckett, L., Carmichael, O., & DeCarli, C. (2014). White matter hyperintensities are associated with visual search behavior independent of generalized slowing in aging. *Neuropsychologia,**52*(1), 93–101. 10.1016/j.neuropsychologia.2013.10.011 24183716 10.1016/j.neuropsychologia.2013.10.011 PMC3924853

[CR78] Madden, D. J., Jain, S., Monge, Z. A., Cook, A. D., Lee, A., Huang, H., Howard, C. M., & Cohen, J. R. (2020). Influence of structural and functional brain connectivity on age-related differences in fluid cognition. *Neurobiology of Aging,**96*, 205–222. 10.1016/j.neurobiolaging.2020.09.010 33038808 10.1016/j.neurobiolaging.2020.09.010 PMC7722190

[CR79] Madden, D. J., Parks, E. L., Tallman, C. W., Boylan, M. A., Hoagey, D. A., Cocjin, S. B., Packard, L. E., Johnson, M. A., Chou, Y., & hui, Potter, G. G., Chen, N. kuei, Siciliano, R. E., Monge, Z. A., Honig, J. A., & Diaz, M. T. (2017). Sources of disconnection in neurocognitive aging: Cerebral white-matter integrity, resting-state functional connectivity, and white-matter hyperintensity volume. *Neurobiology of Aging,**54*, 199–213. 10.1016/j.neurobiolaging.2017.01.027 28389085 10.1016/j.neurobiolaging.2017.01.027 PMC5401777

[CR80] McIntosh, A. R., & Lobaugh, N. J. (2004). Partial least squares analysis of neuroimaging data: Applications and advances. *NeuroImage,**23*(SUPPL. 1), S250–S263. 10.1016/j.neuroimage.2004.07.020 15501095 10.1016/j.neuroimage.2004.07.020

[CR81] Merenstein, J. L., Mullin, H. A., & Madden, D. J. (2023). Age-related differences in frontoparietal activation for target and distractor singletons during visual search. *Attention, Perception, & Psychophysics,**85*, 749–768. 10.3758/s13414-022-02640-x 10.3758/s13414-022-02640-x PMC10066832 36627473

[CR82] Mill, R. D., Ito, T., & Cole, M. W. (2017). From connectome to cognition: The search for mechanism in human functional brain networks. *NeuroImage,**160*, 124–139. 10.1016/j.neuroimage.2017.01.060 28131891 10.1016/j.neuroimage.2017.01.060 PMC5529276

[CR83] Mišić, B., Betzel, R. F., Nematzadeh, A., Goñi, J., Griffa, A., Hagmann, P., Flammini, A., Ahn, Y.-Y., & Sporns, O. (2015). Cooperative and competitive spreading dynamics on the human connectome. *Neuron,**86*(6), 1518–1529. 10.1016/J.NEURON.2015.05.035 26087168 10.1016/j.neuron.2015.05.035

[CR84] Murray, M. E., Senjem, M. L., Petersen, R. C., Hollman, J. H., Preboske, G. M., Weigand, S. D., Knopman, D. S., Ferman, T. J., Dickson, D. W., & Jack, C. R. (2010). Functional impact of white matter hyperintensities in cognitively normal elderly subjects. *Archives of Neurology,**67*(11), 1379–1385. 10.1001/archneurol.2010.280 21060015 10.1001/archneurol.2010.280 PMC3025610

[CR85] Oldham, S., & Fornito, A. (2019). The development of brain network hubs. *Developmental Cognitive Neuroscience,**36*, 100607. 10.1016/j.dcn.2018.12.005 30579789 10.1016/j.dcn.2018.12.005 PMC6969262

[CR86] Peng, S. L., Chen, X., Li, Y., Rodrigue, K. M., Park, D. C., & Lu, H. (2018). Age-related changes in cerebrovascular reactivity and their relationship to cognition: a four-year longitudinal study. *NeuroImage*, 174, 257–262. ://doi.org/10.1016/j.neuroimage.2018.03.033 10.1016/j.neuroimage.2018.03.033 PMC5949266 29567504

[CR87] Pessoa, L. (2018). Emotion and the interactive brain: Insights from comparative neuroanatomy and complex systems. *Emotion Review,**10*(3), 204–216. 10.1177/1754073918765675 31537985 10.1177/1754073918765675 PMC6752744

[CR88] Quandt, F., Fischer, F., Schröder, J., Heinze, M., Lettow, I., Frey, B. M., Kessner, S. S., Schulz, M., Higgen, F. L., Cheng, B., Gerloff, C., & Thomalla, G. (2020). Higher white matter hyperintensity lesion load is associated with reduced long-range functional connectivity. *Brain Communications, 2*(2). 10.1093/braincomms/fcaa111 10.1093/braincomms/fcaa111 PMC7585696 33134915

[CR89] R Core Team. (2019). A language and environment for statistical computing. In R Foundation for Statistical Computing (Vol. 2, p. https://www.R--project.org).

[CR90] Raz, N., Lindenberger, U., Rodrigue, K. M., Kennedy, K. M., Head, D., Williamson, A., Dahle, C., Gerstorf, D., & Acker, J. D. (2005). Regional brain changes in aging healthy adults: General trends, individual differences and modifiers. *Cerebral Cortex,**15*(11), 1676–1689. 10.1093/cercor/bhi044 15703252 10.1093/cercor/bhi044

[CR91] Raz, N., Yang, Y. Q., Rodrigue, K. M., Kennedy, K. M., Lindenberger, U., & Ghisletta, P. (2012). White matter deterioration in 15 months: Latent growth curve models in healthy adults. *Neurobiology of Aging,**33*(2), 429.e1-429.e5. 10.1016/j.neurobiolaging.2010.11.018 21194799 10.1016/j.neurobiolaging.2010.11.018 PMC3131417

[CR92] Reitan RM. Trail Making Test: Manual for administration and scoring. Tucson, AZ: Reitan Neuropsychology Laboratory; 1992.

[CR93] Reuter-Lorenz, P. A., & Park, D. C. (2014). How does it STAC up? Revisiting the scaffolding theory of aging and cognition. *Neuropsychology Review,**24*(3), 355–370. 10.1007/s11065-014-9270-9 25143069 10.1007/s11065-014-9270-9 PMC4150993

[CR94] Rudolph, M. D., Graham, A. M., Feczko, E., Miranda-Dominguez, O., Rasmussen, J. M., Nardos, R., Entringer, S., Wadhwa, P. D., Buss, C., & Fair, D. A. (2018). Maternal IL-6 during pregnancy can be estimated from newborn brain connectivity and predicts future working memory in offspring. *Nature Neuroscience,**21*(5), 765–772. 10.1038/s41593-018-0128-y 29632361 10.1038/s41593-018-0128-y PMC5920734

[CR95] Rudolph, M. D., Miranda-Domínguez, O., Cohen, A. O., Breiner, K., Steinberg, L., Bonnie, R. J., Scott, E. S., Taylor-Thompson, K., Chein, J., Fettich, K. C., Richeson, J. A., Dellarco, D. V., Galván, A., Casey, B. J., & Fair, D. A. (2017). At risk of being risky: The relationship between “brain age” under emotional states and risk preference. *Developmental Cognitive Neuroscience,**24*, 93–106. 10.1016/j.dcn.2017.01.010 28279917 10.1016/j.dcn.2017.01.010 PMC5849238

[CR96] Sadiq, M. U., Langella, S., Giovanello, K. S., Mucha, P. J., & Dayan, E. (2021). Accrual of functional redundancy along the lifespan and its effects on cognition. *NeuroImage,**229*, 117737. 10.1016/j.neuroimage.2021.117737 33486125 10.1016/j.neuroimage.2021.117737 PMC8022200

[CR97] Salat, D. H. (2011). The declining infrastructure of the aging brain. *Brain Connectivity,**1*(4), 279–293. 10.1089/brain.2011.0056 22432418 10.1089/brain.2011.0056 PMC3621330

[CR98] Salthouse, T. A. (1992). What do adult age differences in the Digit Symbol Substitution Test reflect? *Journals of Gerontology, 47*(3). 10.1093/geronj/47.3.P121 10.1093/geronj/47.3.p121 1573192

[CR99] Salthouse, T. A. (1996). The processing-speed theory of adult age differences in cognition. *Psychological Review,**103*(3), 403–428. 10.1037/0033-295X.103.3.403 8759042 10.1037/0033-295x.103.3.403

[CR100] Salthouse, T. A. (2011a). All data collection and analysis methods have limitations: Reply to Rabbitt (2011) and Raz and Lindenberger (2011). *Psychological Bulletin,**137*(5), 796–799. 10.1037/A0024843 21859180 10.1037/a0024843 PMC3160711

[CR101] Salthouse, T. A. (2011b). Neuroanatomical substrates of age-related cognitive decline. *Psychological Bulletin,**137*(5), 753–784. 10.1037/a0023262 21463028 10.1037/a0023262 PMC3132227

[CR102] Salthouse, T. A., & Nesselroade, J. R. (2002). An examination of the Hofer and Sliwinski evaluation. *Gerontology,**48*(1), 18–21. 10.1159/000048919 11844925 10.1159/000048919

[CR103] Sang, L., Qin, W., Liu, Y., Han, W., Zhang, Y., Jiang, T., & Yu, C. (2012). Resting-state functional connectivity of the vermal and hemispheric subregions of the cerebellum with both the cerebral cortical networks and subcortical structures. *NeuroImage,**61*(4), 1213–1225. 10.1016/j.neuroimage.2012.04.011 22525876 10.1016/j.neuroimage.2012.04.011

[CR104] Saults, J. S., & Cowan, N. (2007). A central capacity limit to the simultaneous storage of visual and auditory arrays in working memory. *Journal of Experimental Psychology: General,**136*(4), 663–684. 10.1037/0096-3445.136.4.663 17999578 10.1037/0096-3445.136.4.663 PMC2621445

[CR105] Sherman, S. M., & Guillery, R. W. (2002). The role of the thalamus in the flow of information to the cortex. *Philosophical Transactions of the Royal Society b: Biological Sciences,**357*(1428), 1695–1708. 10.1098/rstb.2002.1161 10.1098/rstb.2002.1161 PMC1693087 12626004

[CR106] Shi, Y., & Wardlaw, J. M. (2016). Update on cerebral small vessel disease: A dynamic whole-brain disease. *Stroke and Vascular Neurology,**1*(3), 83–92. 10.1136/svn-2016-000035 28959468 10.1136/svn-2016-000035 PMC5435198

[CR107] Shine, J. M. (2019). Neuromodulatory Influences on integration and segregation in the brain. *Trends in Cognitive Sciences,**23*(7), 572–583. 10.1016/j.tics.2019.04.002 31076192 10.1016/j.tics.2019.04.002

[CR108] Smith, R. E., Tournier, J. D., Calamante, F., & Connelly, A. (2013). SIFT: Spherical-deconvolution informed filtering of tractograms. *NeuroImage,**67*, 298–312. 10.1016/j.neuroimage.2012.11.049 23238430 10.1016/j.neuroimage.2012.11.049

[CR109] Smith, S. M., Bannister, P., Beckmann, C., & Brady, M. (2001). FSL: New tools for functional and structural brain image analysis. *NeuroImage,**13*, 2001.

[CR110] Sporns, O. (2014). Contributions and challenges for network models in cognitive neuroscience. *Nature Neuroscience,**17*(5), 652–660. 10.1038/nn.3690 24686784 10.1038/nn.3690

[CR111] Taylor, A. N. W., Kambeitz-Ilankovic, L., Gesierich, B., Simon-Vermot, L., Franzmeier, N., Araque Caballero, M., Müller, S., Hesheng, L., Ertl-Wagner, B., Bürger, K., Weiner, M. W., Dichgans, M., Duering, M., & Ewers, M. (2017). Tract-specific white matter hyperintensities disrupt neural network function in Alzheimer’s disease. *Alzheimer’s and Dementia,**13*(3), 225–235. 10.1016/j.jalz.2016.06.2358 27432800 10.1016/j.jalz.2016.06.2358 PMC5319922

[CR112] Thiebaut de Schotten, M., Foulon, C., & Nachev, P. (2020). Brain disconnections link structural connectivity with function and behaviour. *Nature Communications,**11*(1), 1–8. 10.1038/s41467-020-18920-9 10.1038/s41467-020-18920-9 PMC7547734 33037225

[CR113] Tononi, G., Sporns, O., & Edelman, G. M. (1999). Measures of degeneracy and redundancy in biological networks. *Proceedings of the National Academy of Sciences of the United States of America,**96*(6), 3257–3262. 10.1073/pnas.96.6.3257 10077671 10.1073/pnas.96.6.3257 PMC15929

[CR114] Tournier, J. D., Calamante, F., & Connelly, A. (2012). MRtrix: Diffusion tractography in crossing fiber regions. *International Journal of Imaging Systems and Technology,**22*(1), 53–66. 10.1002/ima.22005

[CR115] Tournier, J. D., Smith, R., Raffelt, D., Tabbara, R., Dhollander, T., Pietsch, M., Christiaens, D., Jeurissen, B., Yeh, C. H., & Connelly, A. (2019). MRtrix3: A fast, flexible and open software framework for medical image processing and visualisation. *NeuroImage,**202*, 116137. 10.1016/j.neuroimage.2019.116137 31473352 10.1016/j.neuroimage.2019.116137

[CR116] Tuladhar, A. M., van Dijk, E., Zwiers, M. P., van Norden, A. G. W., de Laat, K. F., Shumskaya, E., Norris, D. G., & de Leeuw, F. E. (2016). Structural network connectivity and cognition in cerebral small vessel disease. *Human Brain Mapping,**37*(1), 300–310. 10.1002/hbm.23032 26466741 10.1002/hbm.23032 PMC6867512

[CR117] Valdés Hernández, M. C., Piper, R. J., Bastin, M. E., Royle, N. A., Muñoz Maniega, S., Aribisala, B. S., Murray, C., Deary, I. J., & Wardlaw, J. M. (2014). Morphologic, distributional, volumetric, and intensity characterization of periventricular hyperintensities. *American Journal of Neuroradiology,**35*(1), 55–62. 10.3174/ajnr.A3612 23811980 10.3174/ajnr.A3612 PMC7966482

[CR118] Varela, C. (2014). Thalamic neuromodulation and its implications for executive networks. *Frontiers in Neural Circuits, 8*. 10.3389/fncir.2014.00069 10.3389/fncir.2014.00069 PMC4068295 25009467

[CR119] Wechsler, D. (1997). *Wechsler Adult Intelligence Scale* (3rd ed.). The Psychological Corporation.

[CR120] Wen, W., & Sachdev, P. (2004). The topography of white matter hyperintensities on brain MRI in healthy 60- to 64-year-old individuals. *NeuroImage,**22*(1), 144–154. 10.1016/j.neuroimage.2003.12.027 15110004 10.1016/j.neuroimage.2003.12.027

[CR121] Wig, G. S. (2017). Segregated systems of human brain networks. *Trends in Cognitive Sciences,**21*(12), 981–996. 10.1016/j.tics.2017.09.006 29100737 10.1016/j.tics.2017.09.006

[CR122] Yang, D., Huang, L., Luo, C., Li, M., Qin, R., Ma, J., Shao, P., Xu, H., Zhang, B., Xu, Y., & Zhang, M. (2020). Impaired structural network properties caused by white matter hyperintensity related to cognitive decline. *Frontiers in Neurology, 11*. 10.3389/fneur.2020.00250 10.3389/fneur.2020.00250 PMC7186334 32373044

[CR123] Yeh, C. H., Smith, R. E., Liang, X., Calamante, F., & Connelly, A. (2016). Correction for diffusion MRI fibre tracking biases: The consequences for structural connectomic metrics. *NeuroImage,**142*, 150–162. 10.1016/j.neuroimage.2016.05.047 27211472 10.1016/j.neuroimage.2016.05.047

[CR124] Yeo, B. T., Krienen, F. M., Sepulcre, J., Sabuncu, M. R., Lashkari, D., Hollinshead, M., Roffman, J. L., Smoller, J. W., Zöllei, L., Polimeni, J. R., Fisch, B., Liu, H., & Buckner, R. L. (2011). The organization of the human cerebral cortex estimated by intrinsic functional connectivity. *Journal of Neurophysiology,**106*(3), 1125–1165. 10.1152/jn.00338.2011 21653723 10.1152/jn.00338.2011 PMC3174820

[CR125] Young, V. G., Halliday, G. M., & Kril, J. J. (2008). Neuropathologic correlates of white matter hyperintensities. *Neurology,**71*(11), 804–811. 10.1212/01.wnl.0000319691.50117.54 18685136 10.1212/01.wnl.0000319691.50117.54

[CR126] Ystad, M., Hodneland, E., Adolfsdottir, S., Haász, J., Lundervold, A. J., Eichele, T., & Lundervold, A. (2011). Cortico-striatal connectivity and cognition in normal aging: A combined DTI and resting state fMRI study. *NeuroImage,**55*(1), 24–31. 10.1016/J.NEUROIMAGE.2010.11.016 21073962 10.1016/j.neuroimage.2010.11.016

[CR127] Zhang, Y., Brady, M., & Smith, S. (2001). Segmentation of brain MR images through a hidden Markov random field model and the expectation-maximization algorithm. *IEEE Transactions on Medical Imaging,**20*(1), 45–57. 10.1109/42.906424 11293691 10.1109/42.906424

