# Supplementary material for: Distributed associations among white matter hyperintensities and structural brain networks with fluid cognition in healthy aging
Source: Cogn Affect Behav Neurosci. 2024 Sep 20;24(6):1121–40. doi: 10.3758/s13415-024-01219-3 (PMC11525275; doi:10.3758/s13415-024-01219-3)
Supplement: Supplementary file 1 — Supplementary file1 (DOCX 3059 KB) [file 13415_2024_1219_MOESM1_ESM.docx]

# Supplementary Material

# Supplementary Methods

## White Matter Hyperintensity Classification and Subcortical Volume Estimation

In addition to assessing global WMH, we delineated arterial and tract-specific derivations to test our hypothesis that WMH-associated age-related disconnection of structural brain networks involves the disruption of subcortical-cortical pathways. As described in (Jiang et al., 2018), the UBO toolbox applies both arterial and tract-specific masks (see Figure S1) to every individual’s WMH mask in DARTEL space. Arterial territories were manually delineated on a single human brain imaged with computed tomography and MRI scans (see Wen and Sachdev, 2004). Arterial derivations included: anterior artery callosal (AAC), anterior artery hemisphere (AAH), middle artery hemisphere (MAH), anterior artery medial lenticulostriate (AAML), middle artery - lateral lenticulostriate (MALL), posterior artery thalamic and midbrain perforators (PATMP), posterior artery hemisphere (PAH), and the posterior artery callosal (PAC). Tract-specific derivations were estimated for several white matter tracts via the Johns Hopkins University (JHU) white-matter tractography atlas (Horn and Blankenburg, 2016). Tracts included: anterior thalamic radiations (ATR), corticospinal tract (CST), cingulate bundle 1 (CING1), cingulate bundle 2 (CING2) forceps major (ForcepsMajor), forceps minor (ForcepsMinor), inferior frontal occipital fasciculus (IFOF), inferior longitudinal fasciculus (ILF), uncinate fasciculus (UF), superior longitudinal fasciculus (SLF), and the temporal extension of the superior longitudinal fasciculus (SLF-TEMP).

**Figure S1. Arterial and tract-based WMH masks utilized by the UBO Detector**


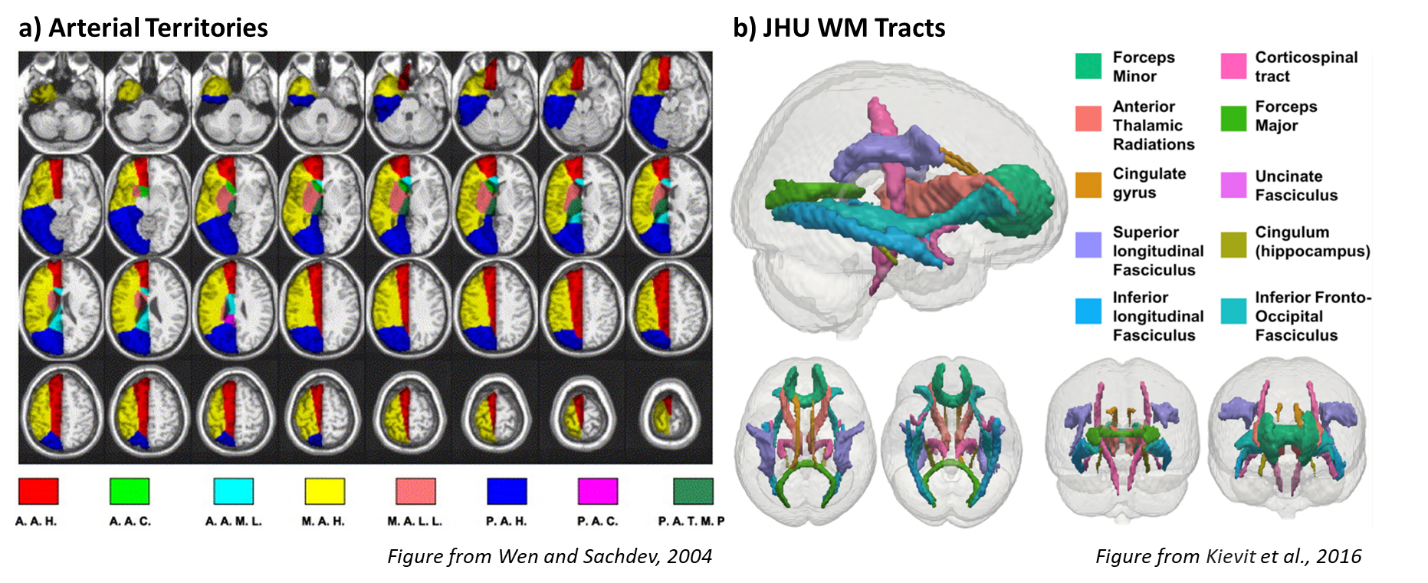


Abbreviations: Arterial derivations included: anterior artery callosal (AAC), anterior artery hemisphere (AAH), middle artery hemisphere (MAH), anterior artery medial lenticulostriate (AAML), middle artery - lateral lenticulostriate (MALL), posterior artery thalamic and midbrain perforators (PATMP), posterior artery hemisphere (PAH), and the posterior artery callosal (PAC).

**Structural Network Topology and Graph Theoretical Metrics**

We calculated the following graph theoretical metrics to assess structural network topology:

***Density.*** Density quantifies the degree of connectedness for a given graph. Global density is defined in Equation 1:

$Global Density=\frac{1}{2} \frac{k}{N^2-N}$ , (1)

where $k$ is the total number of non-zero elements, *N* is the total number of nodes, and the size of the connectivity matrix is *N* x *N*.

***Clustering Coefficient.*** The clustering coefficient measures the degree of connectedness between neighboring nodes. It is the probability that two neighbors of a node are themselves connected, forming a closed triangle. The global clustering coefficient is the average of the clustering coefficient for all nodes in a network and is defined in Equation 2:

$Global Clustering Coefficient= \frac{1}{N}\sum_{i\in N} \frac{1}{k_{i} (k_{i} - 1)}\Sigma_{j,h}\left( {{\hat{w}_{ij}\hat{w}}_{jh}\hat{w}}_{hi} \right)^{1/3}$ , (2)

where $\hat{w}$represents the weight of the connection between node *i* and neighboring nodes j and h, scaled for the maximum weight in a network N (w_ij_ is the weight between nodes i and j, w_jk_ is the weight between nodes j and k, and w_jh_ is the weight between nodes j and h). k_i_ is the degree of node i.

***Modularity.*** Modularity represents the degree to which a system or network can be partitioned into distinct modules, that is, subsets of nodes that are highly interconnected with one another. To partition the whole-brain DWI data into modules we applied the Louvain algorithm (Blondel et al., 2008) from the Brain Connectivity Toolbox (www.brain-connectivity-toolbox.net). Modularity (*Q*) is defined in Equation 3:

$Q=\frac{1}{l^{w}}\sum_{i,j} \left[ w_{ij}-(gamma)\frac{k_{i}^{w}k_{j}^{w}}{l^{w}} \right]\delta\left( m_{i},m_{j} \right)$ , (3)

where $w_{ij}$ is an entry of the weighted connectivity matrix containing the weight (e.g. streamline count) of an edge between nodes *i* and *j,*$k_{i}^{w}$and $k_{j}^{w}$are the sum of the weights *w* of the edges attached to node *i* and *j*, *m_i_* and *m_j_* are the modules to which node *i* or *j* is assigned, and *l^w^* is the total number of weighted edges. *δ* is the Kronecker delta function and gamma is a scaling factor. Specifically, the Louvain algorithm is a greedy-estimation algorithm that attempts to maximize the number of modules based on the resolution parameter gamma. Thus, Q was estimated using gamma values of 1, 1.25, and 1.5 to ensure findings were not dependent on the choice of the scaling factor. As the Louvain algorithm is non-deterministic, we used a consensus clustering approach (Bassett et al., 2013) to determine the optimal number of modules at the individual and whole-group level (Betzel et al., 2017; Lancichinetti & Fortunato, 2012). To do so, Q was computed and averaged over 1000 iterations, and the optimal number of modules was determined by calculating an agreement matrix, for which each cell contained the proportion of times a given pair of nodes was assigned to the same module across all iterations. Consistent with prior work (Madden et al., 2020), agreement matrices were thresholded to retain values of cells for which a node was assigned to the same module at least 50% of the time. In addition to calculating data-driven module assignments, we calculated Q for a module of subcortical regions, and for each of the seven cortical networks (see the previous section, Structural White Matter Connectivity).

***Communicability*.** Communicability is a decentralized measure of information transfer within a given system that quantifies the total number of paths, direct or indirect, that can be traversed by a random walker between any pair of regions (Estrada & Hatano, 2008). Global communicability is the sum communicability for all nodes *n* in a network and is defined in Equation 4:

|  |  |  | $Global Communicability= \sum_{i=1}^{n} \sum_{i=1}^{n} {e^{W}}_{ij}$ | , | (4) |
| --- | --- | --- | --- | --- | --- |

where $e^{W}$ is the matrix exponential of *W*, the weighted connectivity matrix where each cell represents connection weights between a pair of nodes *i* and *j*.

# Supplementary Results

**Linear Relationships Between Age, Fluid Cognition, and WMHs**

As described in the main text (see Main Text; Methods, section 2.4), to compliment analyses assessing linear relationships between age, fluid cognition, and global WMHs (see Results, section 3.1), WMH load was further delineated by arterial and tract-specific derivations (also see Supplementary Methods) to test our hypothesis that the relationship between increased WMHs and decreased connectivity of structural brain networks involves the disruption of subcortical-cortical pathways. Arterial WMH load (Figure 1c, Table S1) was maximal in the bilateral middle cerebral artery and associated lenticulostriate branches, followed by the medial lenticulostriate branches of the anterior artery. This was consistent across thresholds (*k* = .5, *k* = .7). All arterial WMH levels were positively and significantly associated with age after correction for multiple comparisons (*k* = .5: *r* = .25 - .59; *k* = .7: r = .23 – .66; all *p-*FDR < .05) except the left posterior thalamic and midbrain perforators (*k* = .5: *r* = .16, *p*-FDR = .205; *k* = .7: r = .23; *p-*FDR = .071). All arterial WMH levels were negatively and significantly associated with fluid cognition after correction for multiple comparisons (*k* = .5: *r* = -.26 ­– -.60; *k* = .7: r = -.27 – -.66; all *p-*FDR < .05) except the left posterior thalamic and midbrain perforators (*k* = .5: *r* = .16, *p*-FDR = .205; *k* = .7: r = .23; *p-*FDR = .071) and right posterior artery callosal (*k* = .5: *r* = .16, *p*-FDR = .205; *k* = .7: r = .23; *p-*FDR = .071). White matter tract WMH load (Figure 1d, Table S1) was maximal in the anterior thalamic radiations (ATR), followed by the inferior fronto-occipital fasciculus (IFOF), superior longitudinal fasciculus (SLF), and corticospinal tract (CST). All tract-based WMH levels were positively and significantly associated with age after correction for multiple comparisons (*k* = .5: *r* = .32 - .72; *k* = .7: r = .35 - .72; all *p-*FDR< .05). All tract-based WMH levels were negatively and significantly associated with fluid cognition after correction for multiple comparisons (*k* = .5: *r* = -.31 – -.69; *k* = .7: r = -.33 – -.73; all *p-*FDR< .05).

**Table S1. Arterial and tract-based WMHs and correlations with age and fluid cognition**

|  | ***k = .5*** | | | | | | | ***k = .7*** | | | | | | |
| --- | --- | --- | --- | --- | --- | --- | --- | --- | --- | --- | --- | --- | --- | --- |
| **Arterial** | ***Total*** | ***M*** | ***SD*** | ***r (age)*** | ***p^-^*^FDR^** | ***r (cog)*** | ***p^-^*^FDR^** | ***Total*** | ***M*** | ***SD*** | ***r (age)*** | ***p*^-FDR^** | ***r* (cog)** | ***p*^-FDR^** |
| **MAH (R)** | 20915 | 4.15 | 2.08 | 0.59 | **< .001** | -0.56 | **< .001** | 18282 | 3.38 | 2.53 | 0.66 | **< .001** | -0.60 | **< .001** |
| **MALL (L)** | 20061 | 4.35 | 1.85 | 0.58 | **< .001** | -0.60 | **< .001** | 16291 | 3.66 | 2.19 | 0.61 | **< .001** | -0.62 | **< .001** |
| **MAH (L)** | 17273 | 3.91 | 2.09 | 0.48 | **< .001** | -0.47 | **< .001** | 14256 | 3.03 | 2.44 | 0.46 | **< .001** | -0.44 | **< .001** |
| **MALL (R)** | 15538 | 4.35 | 1.43 | 0.52 | **< .001** | -0.52 | **< .001** | 12288 | 3.22 | 2.2 | 0.54 | **< .001** | -0.55 | **< .001** |
| **AAML (R)** | 9494 | 4.52 | 0.91 | 0.51 | **< .001** | -0.48 | **< .001** | 7317 | 3.93 | 1.33 | 0.36 | **0.004** | -0.39 | **0.001** |
| **AAML (L)** | 6949 | 3.97 | 1.30 | 0.51 | **< .001** | -0.53 | **< .001** | 4917 | 3.18 | 1.69 | 0.42 | **< .001** | -0.4 | **< .001** |
| **AAC (L)** | 5660 | 3.36 | 1.55 | 0.57 | **< .001** | -0.56 | **< .001** | 3976 | 1.83 | 2.14 | 0.64 | **< .001** | -0.66 | **< .001** |
| **AAH (R)** | 6045 | 3.49 | 1.34 | 0.53 | **< .001** | -0.52 | **< .001** | 3928 | 2.06 | 1.88 | 0.52 | **< .001** | -0.51 | **< .001** |
| **PAH (L)** | 4350 | 1.95 | 2.00 | 0.47 | **< .001** | -0.56 | **< .001** | 3834 | 1.32 | 1.94 | 0.48 | **< .001** | -0.55 | **< .001** |
| **AAH (L)** | 4036 | 2.71 | 1.63 | 0.56 | **< .001** | -0.49 | **< .001** | 3071 | 1.31 | 1.89 | 0.51 | **< .001** | -0.53 | **< .001** |
| **AAC (R)** | 5137 | 3.49 | 1.45 | 0.40 | **0.001** | -0.38 | **0.002** | 2832 | 1.66 | 1.96 | 0.50 | **< .001** | -0.53 | **< .001** |
| **PAH (R)** | 2828 | 2.27 | 2.13 | 0.57 | **< .001** | -0.57 | **< .001** | 2082 | 1.73 | 2.22 | 0.55 | **< .001** | -0.51 | **< .001** |
| **PATMP (L)** | 2433 | 2.90 | 1.14 | 0.16 | 0.205 | -0.08 | 0.555 | 1215 | 1.35 | 1.6 | 0.23 | 0.071 | -0.23 | 0.060 |
| **PATMP (R)** | 1991 | 2.56 | 1.50 | 0.3 | **0.018** | -0.31 | **0.013** | 786 | 1.12 | 1.53 | 0.43 | **< .001** | -0.45 | **< .001** |
| **PAC (L)** | 726 | 0.69 | 1.45 | 0.25 | **0.045** | -0.26 | **0.035** | 604 | 0.53 | 1.32 | 0.25 | **0.046** | -0.27 | **0.030** |
| **PAC (R)** | 560 | 0.54 | 1.34 | 0.28 | **0.025** | -0.21 | 0.088 | 489 | 0.51 | 1.27 | 0.29 | **0.023** | -0.2 | 0.110 |
| **Tract-based** | ***Total*** | ***M*** | ***SD*** | ***r (age)*** | ***p^-^*^FDR^** | ***r (cog)*** | ***p^-^*^FDR^** | ***Total*** | ***M*** | ***SD*** | ***r* (age)** | ***p^-^*^FDR^** | ***r* (cog)** | ***p^-^*^FDR^** |
| **ATR** | 1852 | 2.50 | 1.18 | 0.66 | **< .001** | -0.58 | **< .001** | 1287 | 1.72 | 1.42 | 0.65 | **< .001** | -0.65 | **< .001** |
| **SLF** | 787 | 1.19 | 1.40 | 0.64 | **< .001** | -0.64 | **< .001** | 763 | 1.3 | 1.29 | 0.61 | **< .001** | -0.61 | **< .001** |
| **IFOF** | 859 | 1.67 | 1.35 | 0.72 | **< .001** | -0.69 | **< .001** | 627 | 1.04 | 1.39 | 0.74 | **< .001** | -0.73 | **< .001** |
| **CST** | 706 | 1.23 | 1.42 | 0.32 | **0.009** | -0.31 | **0.009** | 538 | 0.89 | 1.3 | 0.35 | **0.004** | -0.33 | **0.010** |
| **Forceps Minor** | 580 | 1.76 | 0.91 | 0.51 | **< .001** | -0.53 | **< .001** | 494 | 1.43 | 1.05 | 0.52 | **< .001** | -0.53 | **< .001** |
| **Forceps Major** | 443 | 1.07 | 1.22 | 0.61 | **< .001** | -0.68 | **< .001** | 365 | 0.86 | 1.2 | 0.58 | **< .001** | -0.62 | **< .001** |
| **SLF-TEMP** | 349 | 0.80 | 1.10 | 0.61 | **< .001** | -0.64 | **< .001** | 348 | 0.7 | 1.11 | 0.67 | **< .001** | -0.68 | **< .001** |
| **Uncinate** | 319 | 1.01 | 1.08 | 0.68 | **< .001** | -0.62 | **< .001** | 213 | 0.73 | 0.96 | 0.54 | **< .001** | -0.55 | **< .001** |
| **ILF** | 236 | 0.84 | 1.02 | 0.62 | **< .001** | -0.62 | **< .001** | 160 | 0.65 | 0.91 | 0.64 | **< .001** | -0.63 | **< .001** |
| **CING1** | 185 | 1.05 | 0.70 | 0.54 | **< .001** | -0.52 | **< .001** | 54 | 0.41 | 0.51 | 0.48 | **< .001** | -0.53 | **< .001** |
| **CING2** | 17 | 0.19 | 0.25 | 0.49 | **< .001** | -0.51 | **< .001** | 10 | 0.11 | 0.2 | 0.51 | **< .001** | -0.54 | **< .001** |

Summary statistics highlight WMH load and linear associations between age, fluid cognition, and WMHs.
*Total* represents the sum of raw values before log transformation. Values are sorted by total load (*k* = .7). All log-transformed WMH volumes (mm^3^) were significantly associated with age except the left PATMP. Abbreviations. M = mean; SD = Standard Deviation; cog = Fluid Cognition, FDR = False Discovery Rate, L = Left; R = Right; Arterial regions: AAH, Anterior Artery Hemisphere; MAH, Middle Artery Hemisphere; AAML, Anterior Artery Medial Lenticulostriate; MALL, Middle Artery - Lateral Lenticulostriate; PATMP, Posterior Artery Thalamic and Midbrain Perforators; PAH, Posterior Artery Hemisphere; PAC, Posterior Artery Callosal. White matter tracts: ATR, anterior thalamic radiations; SLF, superior longitudinal fasciculus; IFOF, inferior frontal-occipital fasciculus; CST, corticospinal tract; ILF, inferior longitudinal fasciculus; CING1, cingulate bundle 1; CING2, cingulate bundle 2; Uncinate, uncinate fasciculus).

## Quality Control Analyses

No significant relationship was found between DWI QC metrics and age or fluid cognition (Table S2), or WMH load (Table S3) (all *p*-FDR > .05).

**Table S2. Associations between QC Metrics and both age and fluid cognition**

|  | **Age** | | | | **Fluid Cognition** | | | |
| --- | --- | --- | --- | --- | --- | --- | --- | --- |
|  | ***r*2** | ***r*2^-adj^** | **RMSE** | ***p*^-FDR^** | ***r*2** | ***r*2^-adj^** | **RMSE** | ***p*^-FDR^** |
| Absolute Motion (mm) | .018 | .003 | 0.122 | .334 | 0.008 | -0.006 | 0.121 | 0.642 |
| Contrast to Noise Ratio | .031 | .017 | 21.200 | .223 | 0.023 | 0.008 | 21.300 | 0.440 |
| Relative Motion (mm) | .037 | .022 | 0.035 | .223 | 0.085 | 0.071 | 0.035 | 0.095 |
| Eddy Distortion | .007 | .008 | 0.064 | .487 | 0.002 | -0.013 | 0.065 | 0.702 |
| Number of Outliers | .040 | .026 | 0.110 | .223 | 0.025 | 0.011 | 0.110 | 0.440 |
| Susceptibility Distortion | .037 | .022 | 0.706 | .223 | 0.006 | -0.009 | 0.717 | 0.642 |

No significant relationship (*p*-FDR > .05) was found between DWI QC metrics and age or fluid cognition (p-FDR > .05). Abbreviations. r2 = r-squared; r2-adj = r2 Adjusted; RMSE = Root Mean Squared Error; FDR = False Discovery Rate.

**Table S3. Associations between QC Metrics and WMH load**

|  | ***k* = .5** | | | | ***k* = .7** | | | |
| --- | --- | --- | --- | --- | --- | --- | --- | --- |
|  | ***r*2** | ***r*2^-adj^** | **RMSE** | ***p*^-FDR^** | ***r*2** | ***r*2^-adj^** | **RMSE** | ***p*^-FDR^** |
| **global WMH** |  |  |  |  |  |  |  |  |
| Absolute Motion (mm) | 0.004 | -0.011 | 0.121 | 0.894 | 0.000 | -0.015 | 0.122 | 0.894 |
| Contrast to Noise Ratio | 0.001 | -0.014 | 21.532 | 0.894 | 0.018 | 0.003 | 21.350 | 0.894 |
| Relative Motion (mm) | 0.001 | -0.014 | 0.036 | 0.894 | 0.001 | -0.014 | 0.036 | 0.894 |
| Eddy Distortion | 0.008 | -0.007 | 0.064 | 0.894 | 0.018 | 0.003 | 0.064 | 0.894 |
| Number of Outliers | 0.102 | 0.088 | 0.106 | 0.168 | 0.032 | 0.018 | 0.110 | 0.894 |
| Susceptibility Distortion | 0.005 | -0.012 | 0.765 | 0.894 | 0.013 | -0.004 | 0.762 | 0.894 |
| **pvWMH** |  |  |  |  |  |  |  |  |
| Absolute Motion (mm) | 0.006 | -0.009 | 0.121 | 0.894 | 0.001 | -0.014 | 0.122 | 0.894 |
| Contrast to Noise Ratio | 0.001 | -0.014 | 21.539 | 0.894 | 0.014 | -0.001 | 21.396 | 0.894 |
| Relative Motion (mm) | 0.000 | -0.015 | 0.036 | 0.946 | 0.004 | -0.011 | 0.036 | 0.894 |
| Eddy Distortion | 0.004 | -0.011 | 0.065 | 0.894 | 0.015 | 0.000 | 0.064 | 0.894 |
| Number of Outliers | 0.137 | 0.124 | 0.104 | 0.078 | 0.045 | 0.031 | 0.109 | 0.848 |
| Susceptibility Distortion | 0.002 | -0.015 | 0.766 | 0.894 | 0.012 | -0.005 | 0.763 | 0.894 |
| **dWMH** |  |  |  |  |  |  |  |  |
| Absolute Motion (mm) | 0.002 | -0.014 | 0.122 | 0.894 | 0.003 | -0.012 | 0.121 | 0.894 |
| Contrast to Noise Ratio | 0.005 | -0.010 | 21.492 | 0.894 | 0.000 | -0.015 | 21.543 | 0.894 |
| Relative Motion (mm) | 0.009 | -0.006 | 0.036 | 0.894 | 0.017 | 0.003 | 0.036 | 0.894 |
| Eddy Distortion | 0.010 | -0.005 | 0.064 | 0.894 | 0.034 | 0.020 | 0.064 | 0.894 |
| Number of Outliers | 0.053 | 0.039 | 0.109 | 0.829 | 0.010 | -0.005 | 0.111 | 0.894 |
| Susceptibility Distortion | 0.001 | -0.016 | 0.767 | 0.894 | 0.001 | -0.016 | 0.767 | 0.894 |

No significant relationship (p-FDR > .05) was found between DWI QC metrics and age or fluid cognition (p-FDR > .05). Abbreviations. FDR = false discovery rate; r2 = r-squared; r2-adj = r-squared-adjusted; RMSE = root mean squared error; WMH = white matter hyperintensity; pvWMH = periventricular white matter hyperintensity; dWMH = deep white matter hyperintensity.

**Prediction Analyses**

***Subnetwork Prediction.*** Subnetwork prediction analyses (see Main text Methods, section 2.8.2 and Results, section 3.2.2) were repeated to ensure findings were not specific to the choice of functional or anatomical parcellation. Specifically, random resampling was used to assess the relative significance of subcortical-subcortical and subcortical-cortical models compared to a random null distribution, here, using *anatomical labels* (e.g., lobes) provided by the Brainnetome Atlas. Cortical modules included frontal, insular, limbic, parietal, and temporal lobes. Results are largely comparable to subnetwork prediction analyses based on Yeo 7 modules as presented in the main text (see Results, section 3.2.2). Specifically, models containing connections between subcortical regions and frontal and parietal modules were relatively more predictive than models containing connections between subcortical regions and insular, occipital, and temporal lobes (Table S4).

## Table S4. PLSR Permutation Test Effect Sizes using Anatomically Defined Modules

| **A. Subcortical-Subcortical** | ***k* = .7** | | | | |
| --- | --- | --- | --- | --- | --- |
|  | **KS** | ***d*** | **Med** | **CI-L** | **CI-H** |
|  | 0.97 | 3.12 | 0.67 | 0.38 | 0.84 |
| **B. Subcortical-Cortical** | **KS** | ***d*** | **Med** | **CI-L** | **CI-H** |
|  |  |  |  |  |  |
| **Frontal-Subcortical** | 0.94 | 2.65 | 0.60 | 0.26 | 0.81 |
| **Limbic-Subcortical** | 0.91 | 2.52 | 0.51 | 0.07 | 0.78 |
| **Parietal-Subcortical** | 0.89 | 2.36 | 0.59 | 0.20 | 0.78 |
| **Occipital-Subcortical** | 0.89 | 2.38 | 0.55 | 0.19 | 0.78 |
| **Insular-Subcortical** | 0.83 | 1.96 | 0.59 | 0.14 | 0.81 |
| **Temporal -Subcortical** | 0.75 | 1.65 | 0.44 | 0.00 | 0.72 |

**Network Analyses**

**Assessing linear relationships between global WMH and graph theoretical estimates summarizing structural network topology in older adults.** When controlling for age and fluid cognition across the entire sample, there were no significant relationships between global WMH and structural network topology (see Main text; Results, section 3.3). Given the relevance of age in our sample, we thus conducted supplementary analyses only in participants above the age of 45 years (n = 34, 45 – 78 years, mean age = 61.88 years). In the subsample of middle and older-aged adults, we found that modularity (r^2^ = .12, *p* = .049), but not global clustering coefficient, global communicability, or global density was positively associated with greater WMH load (all *p-fdr* < .05; Figure S2).


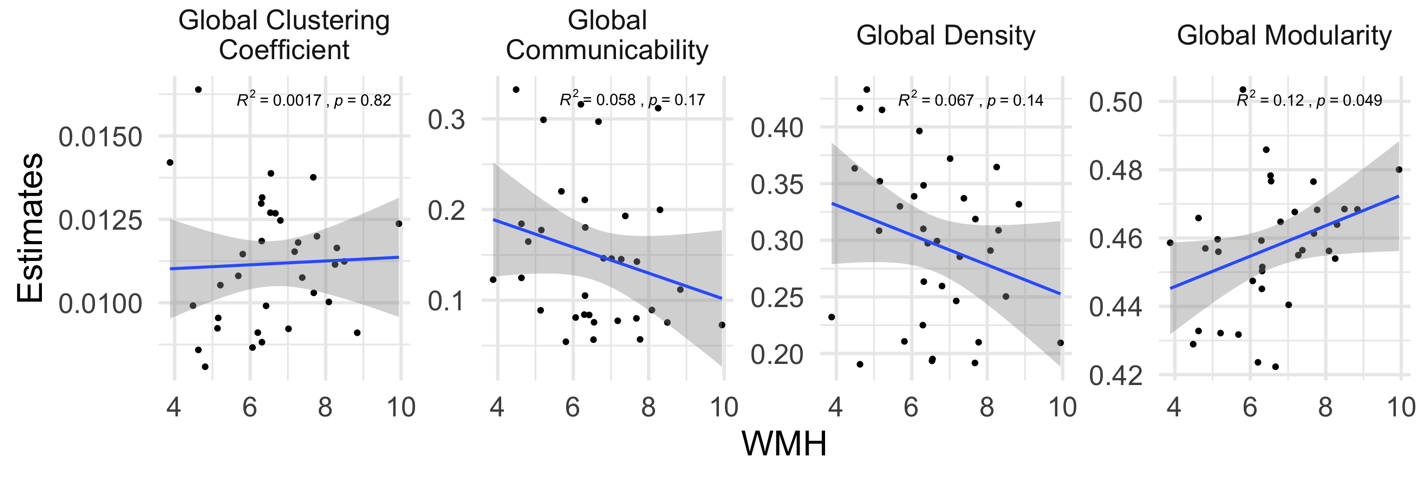


**Figure S2. Results of linear regression analysis assessing association between graph theoretical summary estimates of structural network topology and global WMH.** In middle and older-aged adults (ages 45-78), higher modularity is positively associated with greater WMH load.

**Post-Hoc Exploratory Cognitive Domain and Test-Specific Sensitivity Analyses**

*Linear Relationships Between Age, Factor Scores, and Individual Performance Metrics.* As described in the main text (see Main Text; Results, section 3.1), age was significantly negatively associated with the general composite of fluid cognition (*r* = -.859, *p* < .0001) derived via factor analysis using scores obtained on a battery of 12 cognitive tests as described in the main text (see Main Text; Methods, section 2.2 for details). As shown in Figure S3, age was also significantly negatively associated with (1) individual factor scores (perceptual speed: *r* = -.81; executive function: *r* = -.80; memory: *r* = -.64; all *p* < .001) (Figure S3, top row) and, (2) with performance on the NIH Toolbox Dimensional Change Card Sort Test (*r* = -.66; p < .001) and NIH Toolbox Picture Sequence Memory Test (*r* = -.66; p < .001) and NIH Toolbox Pattern Comparison Test speed score (*r* = -.78, p < .001) (Figure S3, bottom row). Additionally, all cognitive measures assessed were significantly positively associated with one another (*r* = .51-.96; all *p* < .001).


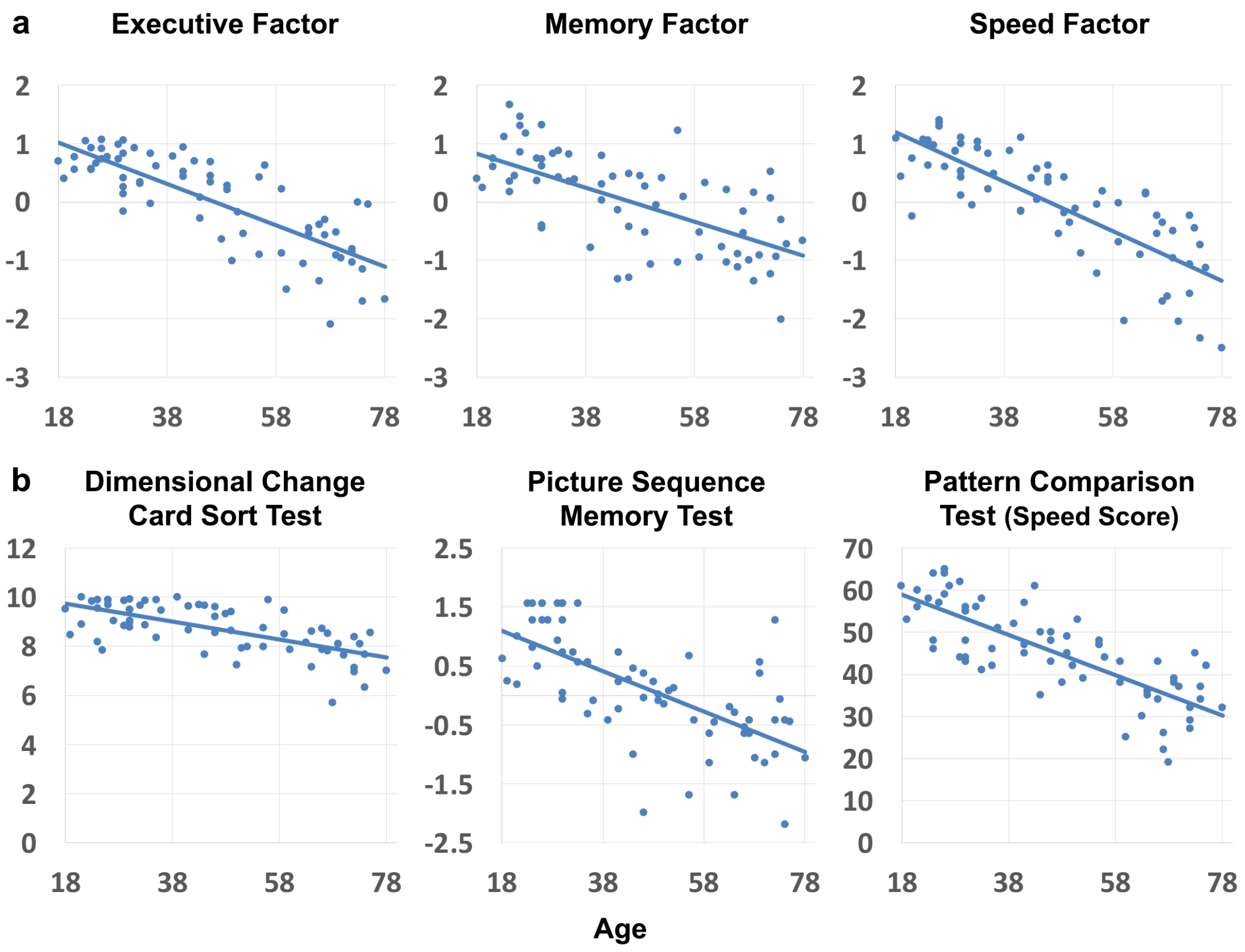


**Figure S3 Relationships Between Age, Cognitive Factors, and Individual Performance Measures**. a) There were significant negative linear associations between age and each of the cognitive domain-specific factor scores derived using a factor analytic approach as described in the main text (see Methods, section 2.2). b) There were significant negative linear associations between age and test-specific performance scores on the NIH Toolbox Dimensional Change Card Sort Test and NIH Toolbox Picture Sequence Memory Test, and the NIH Toolbox Pattern Comparison Test speed score.

**Whole-Brain Prediction Analyses Assessing Three Indices of Cognitive Functioning from the NIH Toolbox.**

Primary analyses assessing the relationship between an individual’s structural connectome and age, global WMH (*k* = .7), and fluid cognition were repeated using metrics from the NIH toolbox assessments as described above and in the main text (see Methods, section 2.2). 5-fold cross-validated PLSR models using all available structural connections achieved average model accuracies of approximately 98%, 78%, and 86% respectively when assessing performance assessing general cognitive domains on the NIH Toolbox Dimensional Change Card Sort Test (executive function) and the NIH Toolbox Picture Sequence Memory Test (memory), and the NIH Toolbox Pattern Comparison Test speed score (processing speed).

*NIH Toolbox Dimensional Change Card Sort Test.* For the model predicting the covariance between age, global WMH, and performance on the *NIH Toolbox Dimensional Change Card Sort Test*, four components maximized the amount of variance explained (CV-PCTVAR = 98.10) and minimized the mean-squared prediction error in the held-out test set across folds (CV-MSEP = 125.89; baseline MSE = 329.22).

*NIH Toolbox Picture Sequence Memory Test.* For the model predicting the covariance between age, global WMH, and performance on the NIH Toolbox Picture Sequence Memory Test, four components maximized the amount of variance explained (CV-PCTVAR = 78.03) and minimized the mean-squared prediction error in the held-out test set across folds (CV-MSEP = .88; baseline MSE = .83).

*NIH Toolbox Pattern Comparison Test speed score.* For the model predicting the covariance between age, global WMH, and NIH Toolbox Pattern Comparison Test speed score, three components maximized the amount of variance explained (CV-PCTVAR = 85.78) and minimized the mean-squared prediction error in the held-out test set across folds (CV-MSEP = 208.77; baseline MSE = 450.08).

As with the primary cross-validated PLS model predicting fluid cognition, all connections contributed to model performance, and the top predictive connections were distributed across each of the major lobes. The most prominent connections were between pairs of regions within subcortical, frontal, and parietal lobes, and between subcortical regions with the frontal, limbic, and parietal lobes (Figure S4). Overall, predictive features (absolute beta weights) were highly similar between each of the three supplementary models predicting the covariance between age, global WMH, and performance on the *NIH Toolbox Dimensional Change Card Sort Test*, *NIH Toolbox Pattern Comparison Test speed score*, and *NIH Toolbox Picture Sequence Memory Test* respectively (Spearman’s *rho* = .84 - .86; *r* = .69 - .75), and with those obtained in the primary model based on our measure of fluid cognition (Spearman’s *rho* = .85; *r* = .77). In addition to the high collinearity between our cognitive factor scores as described in the main text, we note that the three specific task performance measures are also correlated in our sample (r = .55 - .65), thus this limits our ability to disentangle the relationship between patterns of WMH and specific domains of cognition.


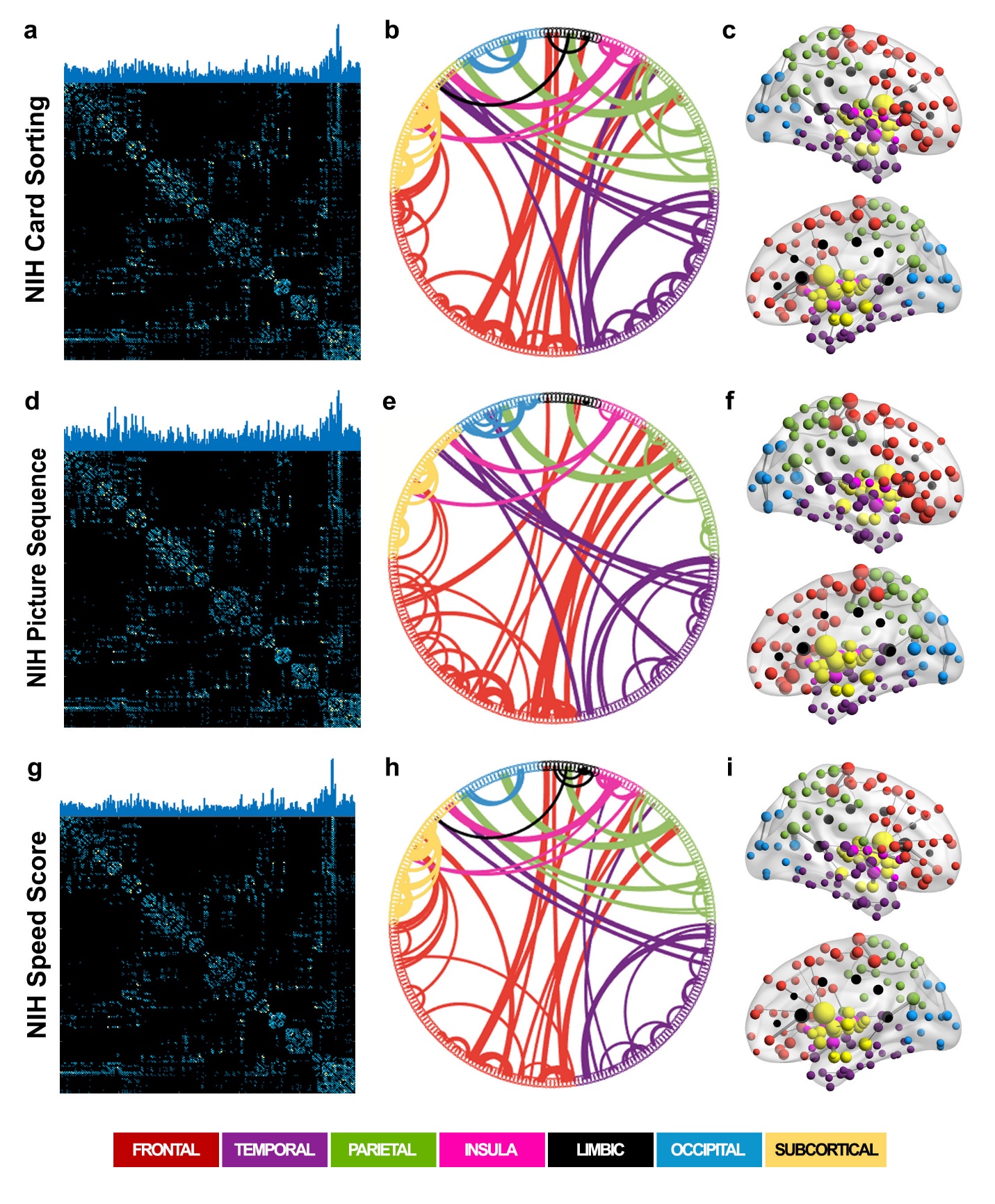


**Figure S4. Results of analysis predicting age, individual NIH cognitive metrics, and global WMH from the whole-brain structural connectome.** Predictive features from the whole-brain PLSR models predicting performance on the NIH Toolbox Dimensional Change Card Sort Test (executive function; top row), NIH Toolbox Picture Sequence Memory Test (memory; middle row), NIH Toolbox Pattern Comparison Test speed score (processing speed; bottom row). Panels (a, d, g) show prediction weights (e.g., absolute beta weights) for all structural connections in matrix form (symmetrical matrix; 246 x 246 regions). Each cell represents a connection between a pair of ROIs. Brighter colors represent connections weighted more heavily in the prediction model. The overall importance of an ROI (sum of prediction weights across a row in the matrix) is shown in the bar plot at the top of the matrix. Colored bars below the matrix represent anatomical labels provided by the Brainnetome atlas (F = Frontal (red); T = Temporal (purple); P = Parietal (green); I = Insular (pink); L = Limbic (black); O = Occipital (blue); S = Subcortical (yellow). b) The top 1% of connections are plotted for visualization purposes. Panels (b, e, h) show connections as edges between regions arranged by lobe according to the anatomical labels provided by the Brainnetome atlas. Panels (c, f, i) show the same connections displayed on a standard brain surface. Lines represent connections between brain regions and are scaled by their prediction weights. ROIs are depicted as spheres and are scaled by the sum of their absolute beta weights. Connections and ROIs are colored according to the Brainnetome atlas labels.
